# Multifunctional Nanostructure RAP‐RL Rescues Alzheimer's Cognitive Deficits through Remodeling the Neurovascular Unit

**DOI:** 10.1002/advs.202001918

**Published:** 2020-12-10

**Authors:** Qian Zhang, Qingxiang Song, Xiao Gu, Mengna Zheng, Antian Wang, Gan Jiang, Meng Huang, Huan Chen, Yu Qiu, Bin Bo, Shanbao Tong, Rong Shao, Binyin Li, Gang Wang, Hao Wang, Yongbo Hu, Hongzhuan Chen, Xiaoling Gao

**Affiliations:** ^1^ Department of Pharmacology and Chemical Biology State Key Laboratory of Oncogenes and Related Genes Shanghai Universities Collaborative Innovation Center for Translational Medicine Shanghai Jiao Tong University School of Medicine 280 South Chongqing Road Shanghai 200025 China; ^2^ School of Biomedical Engineering and Med‐X Research Institute Shanghai Jiao Tong University 800 Dongchuan Road Shanghai 200240 China; ^3^ Department of Neurology & Neuroscience Institute Ruijin Hospital affiliated to Shanghai Jiao Tong University School of Medicine 197 Rui Jin Er Road Shanghai 200025 China; ^4^ Institute of Interdisciplinary Integrative Biomedical Research Shuguang Hospital Shanghai University of Traditional Chinese Medicine 1200 Cailun Road Shanghai 201210 China

**Keywords:** Alzheimer's disease, cerebrovasculature, multifunctional nanostructure, NVU remodeling

## Abstract

Cerebrovascular dysfunction characterized by the neurovascular unit (NVU) impairment contributes to the pathogenesis of Alzheimer's disease (AD). In this study, a cerebrovascular‐targeting multifunctional lipoprotein‐biomimetic nanostructure (RAP‐RL) constituted with an antagonist peptide (RAP) of receptor for advanced glycation end‐products (RAGE), monosialotetrahexosyl ganglioside, and apolipoprotein E3 is developed to recover the functional NVU and normalize the cerebral vasculature. RAP‐RL accumulates along the cerebral microvasculature through the specific binding of RAP to RAGE, which is overexpressed on cerebral endothelial cells in AD. It effectively accelerates the clearance of perivascular A*β*, normalizes the morphology and functions of cerebrovasculature, and restores the structural integrity and functions of NVU. RAP‐RL markedly rescues the spatial learning and memory in APP/PS1 mice. Collectively, this study demonstrates the potential of the multifunctional nanostructure RAP‐RL as a disease‐modifying modality for AD treatment and provides the proof of concept that remodeling the functional NVU may represent a promising therapeutic approach toward effective intervention of AD.

## Graphical abstract

RAP‐RL, a multifunctional lipoprotein‐like nanostructure, which contains RAGE antagonist peptide (RAP), GM1 and ApoE3, is designed to rescue AD cognitive deficits through remodeling the neurovascular unit. RAP‐RL accumulates along the cerebral microvasculature through the specific binding of RAP to RAGE. It decreases neuroinflammation, accelerates the perivascular Aβ clearance, restores the structure and function of the cerebrovasculature system, and efficiently alleviates spatial learning and memory in AD model mice.

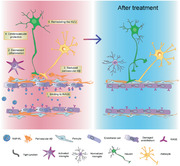

## 1 Introduction

Alzheimer's disease (AD) is the most prevalent senile dementia and becomes one of the most common healthcare challenges. Regardless of substantial endeavor for decades, the development of effective disease‐modifying therapeutics for AD has frequently failed, making the morbidity of AD high static.^[^ ^1^ ^]^ More than 20 compounds which are mainly based on amyloid and tau hypothesis have completed large phase 3, double‐blind, randomized control trials in cohorts of patients at various stages of AD. None of them has demonstrated satisfying efficacy in slowing cognitive decline or improving global brain function.^[^ ^2^ ^]^ Therefore, the necessity of novel therapeutic regime targeting the key factors and restoring normal cellular function in AD is extremely urgent.

Increasing evidence demonstrates that cerebrovascular dysfunction is an early pathological event in AD and contributes significantly to the permanent loss of neuronal function.^[^ ^3^ ^]^ Pathological investigations from 80% of patients diagnosed with AD but without evidence of mixed dementia exhibited pathogenic progression associated with various types of cerebrovascular dysfunction including lacunes, cortical infarcts, cerebral microbleeds, multiple microinfarcts, and intracranial atherosclerosis.^[^ ^4^ ^]^ Of note, the changes in cerebral blood perfusion happen long before clinical symptoms of AD and even before *β*‐amyloid (A*β*) accumulation.^[^ ^3b^ ^]^ Accordingly, cerebrovascular dysfunction has been put forward three decades ago, and two‐hit vascular hypothesis was proposed to demonstrate the important role of cerebrovascular dysfunction in the progression of AD.^[^ ^5^ ^]^ Hit one, the AD risk factors such as aging, hypertension, diabetes, and apolipoprotein E4 (ApoE4) genotype lead to cerebrovascular damage, inducing blood‐brain barrier (BBB) damage and brain perfusion reduction.^[^ ^4^, ^6^ ^]^ Hit two, the BBB impairment increases the intracerebral accumulation of blood‐derived neurotoxic molecules including A*β*, and decreases its clearance, while oligemia further promotes A*β* production and elevates tau phosphorylation.^[^ ^6a^ ^]^ These A*β*‐independent and A*β*‐dependent pathways synergistically lead to synaptic and neuronal dysfunction, which ultimately contribute to the development of dementia. Therefore, effective therapeutics targeting the dysfunctional cerebrovascular network may improve the neurological activity in AD.

In order to treat AD through the modulation of cerebrovascular function, various existing therapeutics modulating cerebrovascular function including 3‐hydroxy‐3‐methyl glutaryl coenzyme A reductase inhibitor simvastatin, angiotensin‐converting enzyme inhibitor enalapril, angiotensin II type 1 receptor antagonist losartan and the peroxisome proliferator‐activated receptor *γ* agonist pioglitazone have been tested.^[^ ^7^ ^]^ However, these efforts eventually failed to alleviate the cognitive deficits in AD patients and AD model mice,^[^ ^7^, ^8^ ^]^ presumably due to the complex structure and function of the cerebrovascular network. As the key component of the cerebrovascular system, neurovascular unit (NVU), composed of endothelial cells, neurons, astrocytes, microglia, pericytes or vascular smooth cells, and basal membrane, plays an important role in neurovascular coupling to ensure the rapid and sufficient energy supply for brain function.^[^ ^6a^ ^]^ In the pathogenesis of AD, NVU is structurally and functionally impaired, which includes loss of coupling among neurons, glial cells and vascular cells, injury of endothelial cells, loss of pericytes, thickening of basal membrane, loose of tight junctions, and destruction of BBB integrity.^[^ ^9^ ^]^ Such alterations of the NVU reduce cerebral blood flow (CBF) and decrease cerebral oxygenation, leading to cerebral hypoxia.^[^ ^3a^ ^]^ The concomitant BBB dysfunction not only reduces the intracerebral transport of glucose and other nutrients,^[^ ^10^ ^]^ but also decreases the removal of excess metabolites,^[^ ^11^ ^]^ thus alters the homeostasis in the central nervous system (CNS). Alterations of clearance pathways also promote the accumulation of molecules such as A*β* and tau, leading to proteinopathies. These evidences suggest that the cerebrovascular dysfunction associated with the NVU changes plays key roles in the progression of AD and in the deterioration of cognitive function.^[^ ^9^, ^12^ ^]^ Accordingly, we hypothesized that multifunctional therapeutics which can target the damaged brain vessels, remediate the multicomponents of the NVU and thus normalize the cerebrovascular system would provide a promising approach to AD treatment.

**Scheme 1:**
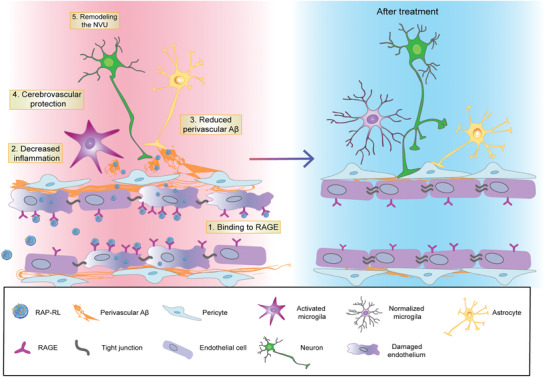
RAP‐RL accumulates along the cerebral microvasculature through the specific binding of RAP to RAGE which is overexpressed on AD cerebral endothelial cells. It decreases neuroinflammation, accelerates the clearance of perivascular A*β*, normalizes the morphology and function of cerebrovasculature, and restores the structural integrity and function of NVU.

To justify the above hypothesis, here we designed a multifunctional lipoprotein‐like nanostructure, which contains RAP, a peptide that can bind to the injured cerebrovasculature through recognition of the receptor for advanced glycation end products (RAGE), and a monosialotetrahexosyl ganglioside (GM1)‐incorporated reconstituted lipoprotein (RL).^[^ ^13^ ^]^ RAGE is overexpressed on the injured cerebrovasculature in both AD patients and AD model mice.^[^ ^14^ ^]^ RAP, a peptide derived from RAGE's nature ligand, S100B, can bind RAGE effectively.^[^ ^15^ ^]^ GM1, an important lipid component in the brain, especially in neurons,^[^ ^16^ ^]^ reduced the damage of brain system caused by trauma and ischemia, promoted the repair of injured neurons and improved the cognition of AD patients following intraventricular injection.^[^ ^13^, ^17^ ^]^ Apolipoprotein E3 (ApoE3), another key component of the lipoprotein‐like nanostructure, acts as an effective anti‐inflammatory molecule.^[^ ^18^ ^]^ Besides, lipidated ApoE3 has been shown to accelerate the clearance of A*β* and suppress the CypA‐nuclear factor kappa B‐matrix metalloproteinase‐9 pathway in pericytes to maintain cerebrovascular integrity.^[^ ^19^ ^]^ Collectively, we proposed that through successful self‐assembly of the multiple key components including RAP, GM1, and ApoE3 in a nanostructure, the obtained nanostructure RAP‐RL might remediate the multicomponents of the NVU and thus normalize the cerebrovascular system (**Scheme** **1**). As expected, RAP‐RL efficiently targeted the injury cerebral microvasculature, regained the integrity and function of NVU and improved cognition in AD model mice, providing proof of concept that rebuilding the functional NVU may give rise to therapeutic promising toward effective treatment of AD.

## 2 Results

### 2.1 Construction and Characterization of RAP‐RL

**Figure 1:**
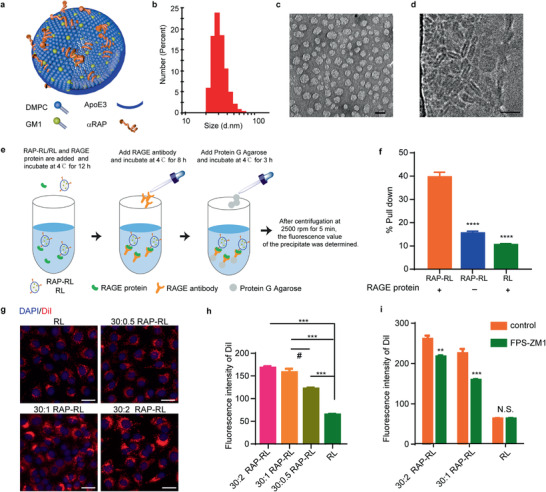
Construction and characterization of RAP‐RL. a) Schematic diagram shows the structure and components of RAP‐RL. b) Size distribution of RAP‐RL measured through DLS. c) Morphology of RAP‐RL observed under TEM after negative staining with phosphotungstic acid (1.5%, w/v). Scale bar, 25 nm. d) Structure of RAP‐RL observed under cryo‐EM. Scale bar, 20 nm. e) Scheme shows RAP‐RL and RAGE protein coimmunoprecipitated by protein G agarose in the presence of RAGE antibody. f) Quantification of DiI‐labeled RAP‐RL (DiI‐RAP‐RL) pulled down by protein G agarose in APP/PS1 mice plasma. g) Cellular uptake of DiI‐RAP‐RL in bEnd.3 cells. h) Quantification of the cellular uptake of DiI‐RAP‐RL in bEnd.3 cells after 6‐h incubation (*n* = 4). Scale bars, 30 µm. i) Cellular uptake of DiI‐RAP‐RL inhibited by RAGE inhibitor FPS‐ZM1. bEnd.3 cells were pretreated with FPS‐ZM1 (5 × 10^−6^ m) for 1 h, and then incubated with RAP‐RL (5 µg mL^−1^ DMPC) for 3 h (*n* = 5). Data represent the mean ± SEM. ^#^ *P* < 0.05, **P < 0.01, ***P < 0.001. i) Student's *t*‐test or f,h) one‐way ANOVA with Tukey's multiple‐comparisons test for group comparisons.

RAP‐RL was created by first preparing liposome with GM1 with 1,2‐dimyristoyl‐*sn*‐glycero‐3‐phosphocholine (DMPC) through the thin film‐hydration method, and then incubating the liposome with *α*‐helix (FAEKFKEAVKDYFAKFWD)‐linked RAP peptide (ELKVLMEKEL) (*α*RAP) and ApoE3 sequentially (**Figure** **1**a) to self‐assemble the lipoprotein‐like nanostructure. The *α*‐helix sequence exhibits an amphipathic helix structure and strong lipid affinity as apolipoprotein,^[^ ^20^ ^]^ ensuring the successful assembling between the lipid membrane and the RAP peptide. Given the nature of the nanoparticles, dynamic light scattering (DLS) (Figure 1b), transmission electron microscopy (TEM) (Figure 1c), and cryo‐electron microscopy (cyro‐EM) (Figure 1d) were employed to substantially characterize their physiochemical properties. RAP‐RL displayed a nanodisc structure with the size 23 ± 2 nm and zeta potential ‐8.8 ± 1.5 mV. Surface plasmon resonance (SPR) analysis demonstrated that the binding affinity constant (*K*_D_) of *α*RAP with RAGE was 1.62 × 10^−9^ m, comparable with that of RAP (2.79 × 10^−9^ m) (Figure S1, Supporting Information). To evaluate the interaction between RAP‐RL and RAGE under physiological conditions, coimmunoprecipitation analysis was performed in APP/PS1 mice plasma with RAGE antibody as the bait protein. 1,10‐Dioctadecyl‐3,3,30,30‐tetramethylindocarbocyanine perchlorate (DiI) (2% to DMPC, w/w) was incorporated into the membrane of RAP‐RL (DiI‐RAP‐RL) and RL (DiI‐RL) for fluorescent labeling (Figure 1e). In the presence of RAGE protein, the levels of DiI‐RAP‐RL pulled down by protein G agarose was 3.87 and 2.59 folds to that of DiI‐RL and DiI‐RAP‐RL in the absence of RAGE, respectively (Figure 1f), indicating that RAP‐RL could efficiently bind to RAGE even in plasma.

As the density of *α*RAP peptide was a key factor influencing the targeting efficiency, the cellular uptake of DiI‐RAP‐RL with various densities of *α*RAP peptide was evaluated in bEnd.3 cells, a mouse brain microvascular endothelial cell line (Figure S2, Supporting Information). The cellular uptake of RAP‐RL increased as the DMPC to *α*RAP molar ratio increased from 30:0.5, 30:1 to 30:2 (Figure 1g,h). In addition, RAGE inhibitor FPS‐ZM1 inhibited the cellular uptake of RAP‐RL (Figure 1i), indicating that RAGE is required and helpful for the cellular uptake of RAP‐RL. To determine if the RAP‐RAGE interaction would work in AD patients, we directly incubated RAP‐RL with brain slices from AD patients, finding that RAP‐RL exhibited higher accumulation along the vessel walls than that in the parenchyma, other than extensively distributed in various cells in the non‐AD brain samples without specific accumulation along the vasculature (Figure S3, Supporting Information). These data indicated that RAP‐RL might efficiently target the injured cerebral micro‐vessels in AD patients.

### 2.2 RAP‐RL Preferentially Binds to the Cerebral Microvasculature in AD Model Mice

**Figure 2:**
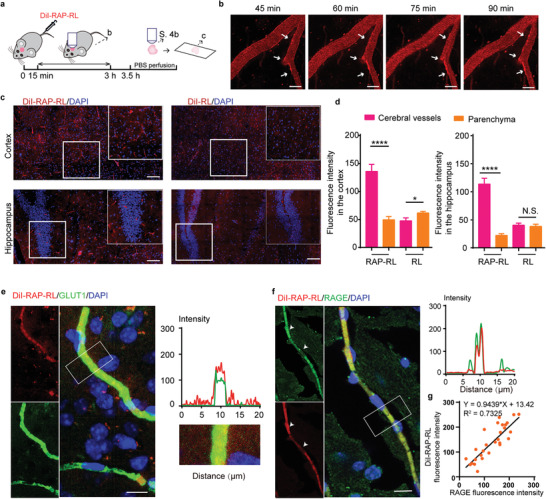
RAP‐RL preferentially binds to the cerebral microvasculature in AD model mice. APP/PS1 mice were treated with DiI‐RAP‐RL (5 mg kg^−1^ DMPC) intravenously. a) Schematic of experimental design. b) Time‐dependent accumulation of DiI‐RAP‐RL along the cerebrovascular wall under multi‐photon microscopy. Scale bars, 50 µm. c) Distribution of DiI‐RAP‐RL and DiI‐ RL in the cortex and hippocampus. Scale bars, 100 µm. d) Quantification of the levels of DiI‐RAP‐RL and DiI‐ RL in cerebral vessels and parenchyma (*n* = 3, 42 ROI per group). e) Left, DiI‐RAP‐RL colocalized with GLUT1 and f) RAGE, both of which exhibit typical blood vessel expression profile. Right, zoom‐in images and its signal intensity profile of DiI‐RAP‐RL (red line) and GLUT1 (green line) or RAGE (green line). Scale bars, 10 µm. g) Fluorescent intensity correlation between RAGE and DiI‐RAP‐RL (*n* = 3, 27 ROI). Data represent the mean ± SEM. *P < 0.01, ****P < 0.0001, Student's *t*‐test for group comparisons. N.S., not significant.

Elevated expression of RAGE was found in the injured cerebral endothelial cells in AD patients and AD model animals,^[^ ^14^, ^21^ ^]^ where the impaired cerebral vasculature located and its cascade occurred. To determine whether RAP‐RL can efficiently target the damaged cerebral microvasculature, DiI‐RAP‐RL or DiI‐RL was injected intravenously to the APP/PS1 mice, respectively (**Figure** **2**a). Under the observation through multiphoton microscopy, RAP‐RL showed specific accumulation along the cerebral vessels as early as 45 min and increased at 90 min after administration (Figure 2b). In contrast, RL did not show such accumulation (Figure S4a, Supporting Information). At 3.5 h after administration, the mice were perfused and the harvested brain was subjected to multiphoton microscopy analysis again. RAP‐RL was also majorly found along the wall of the microvasculature of APP/PS1 mice, while RL did not show adhesion to the vascular walls (Figure S4b, Supporting Information). Consistently, confocal microscopy analysis found that RAP‐RL accumulated along the microvessel walls at the level much higher than that in the parenchyma (Figure 2c,d). Moreover, RAP‐RL was also found well colocalized with GLUT1 (Figure 2e) and RAGE (Figure 2f), both of which exhibit typical cerebral capillary morphological profile. Moreover, the signal intensity of RAP‐RL was positively correlated with the intensity of RAGE (Figure 2g), supporting the hypothesis that RAP‐RL can preferentially bind to the injured cerebral microvasculature.

### 2.3 RAP‐RL Alleviates A*β*‐Induced Tight‐Junction Impairment and Inflammation In Vitro

**Figure 3:**
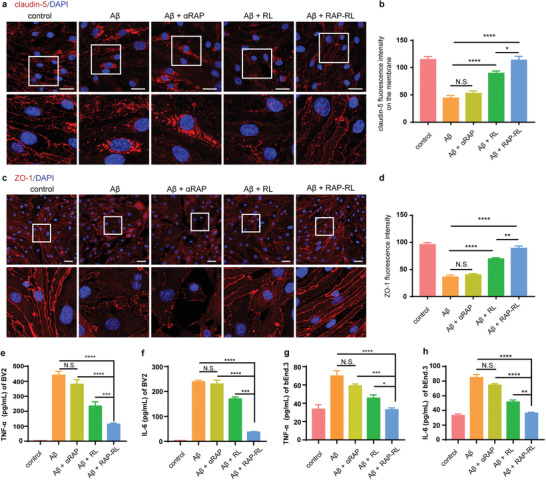
RAP‐RL alleviates A*β*‐induced junction protein impairment and inflammation in vitro. bEnd.3 cells or BV2 cells were pretreated with A*β*_1‐42_‐oligomer (5 × 10^−6^ m) for 18 h, and then incubated with RAP‐RL (containing 6 µg mL^−1^ DMPC), RL (containing 6 µg mL^−1^ DMPC) or *α*RAP (0.92 µg mL^−1^, equally to the amount of aRAP in RAP‐RL) for 18 h. a) Claudin‐5 immunofluorescence (red) in bEnd.3 cells following *α*RAP, RL, or RAP‐RL treatments. DAPI (blue) was used to stain nuclei. Scale bar, 30 µm. b) Quantification of claudin‐5 fluorescence intensity on the cell membrane (*n* = 5). c) ZO‐1 immunofluorescence (red) in bEnd.3 cells following the different treatments. DAPI (blue) was used to stain nuclei. Scale bar, 30 µm. d) Quantification of ZO‐1 fluorescence intensity (*n* = 5). e) Levels of TNF‐*α*, f) IL‐6 in the supernatants of BV2 cells following the *α*RAP, RL, or RAP‐RL treatments (*n* = 5). g) Levels of TNF‐*α*, h) IL‐6 in the supernatants of bEnd.3 cells following the *α*RAP, RL, or RAP‐RL treatments (*n* = 5). Data represent the mean ± SEM. *P < 0.05, **P < 0.01, ***P < 0.001, ****P < 0.0001, N.S., not significant. One‐way ANOVA with Tukey's multiple‐comparisons test for group comparisons.

It has been reported that BBB breakdown in AD could be related to altered endothelial junctions,^[^ ^22^ ^]^ thus we analyzed the expression of tight‐junction proteins in bEnd.3 cells treated with A*β*_1‐42_oligomer to investigate the vascular‐protection ability of RAP‐RL. It was found that following the incubation with A*β*_1‐42_ oligomer (5 × 10^−6^ m) for 18 h, most claudin‐5 in bEnd.3 cells translocated from the cell membrane into the cytoplasm, while RAP‐RL treatment largely reversed such translocation (**Figure** **3**a,b). Moreover, the expression of ZO‐1 on the cell membrane, which was disrupted by A*β*_1‐42_ oligomer, was almost completely reversed after the RAP‐RL treatment (Figure 3c,d). RL also reversed the expression of claudin‐5 and ZO‐1 but in a less extent (Figure 3a–d), while RAP itself did not affect the expression of claudin‐5 and ZO‐1 (Figure 3a–d). Additionally, in the absence of A*β*_1‐42_ oligomer, RAP‐RL (containing 6 to 90 µg mL^−1^ DMPC) demonstrated no effect on the morphology and claudin‐5 and ZO‐1 expression in bEnd.3 cells (Figure S5, Supporting Information), indicating that RAP‐RL did not exert obvious off‐target effects on the non‐injured cerebral microvasculature in this model.

Neuroinflammation is another key hallmark in AD brain.^[^ ^23^ ^]^ The cerebral microvasculature is a participant in a destructive cycle of events where inflammation precedes A*β* deposition and A*β* in turn promotes the release of inflammatory mediators. Moreover, the aggregated A*β* could activate microglia and induce the secretion of proinflammatory cytokines, which further induced A*β* aggregation and neuronal death.^[^ ^24^ ^]^ In this regard, exposure of bEnd.3 and BV2 cells to A*β*_1‐42_‐oligomer could evoke cascade proinflammatory responses, which was used to evaluate the anti‐inflammatory properties of RAP‐RL. As displayed in Figure 3e,f, the supernatant level of TNF‐*α* and IL‐6 significantly increased to 430 pg mL^−1^ and 239 pg mL^−1^ in the A*β*_1‐42_‐ treated BV2 cells. RAP‐RL and RL noticeably reduced the TNF‐*α* production by 73.38% and 46.78%, and reduced IL‐6 levels by 84.28% and 28.67%, respectively. In contrast, RAP itself did not exert a significant effect on the level of TNF‐*α* and IL‐6 (Figure 3e,f). Similarly, RAP‐RL also significantly reduced the level of TNF‐*α* and IL‐6 in the supernatant of those A*β*_1‐42_‐treated bEnd.3 cells, while RAP itself did not exert a significant effect on proinflammatory cytokines production (Figure 3g,h).

### 2.4 RAP‐RL Reduces Perivascular A*β* Depositions in the Brain of APP/PS1 Mice

**Figure 4:**
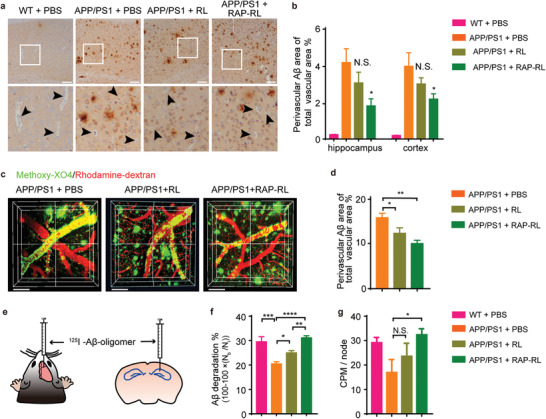
RAP‐RL significantly reduces perivascular A*β* depositions in the brain of APP/PS1 mice. a,b) 13‐month‐old male APP/PS1 mice were treated with RAP‐RL or RL for 4 weeks with the age‐match WT and APP/PS1 mice treated with PBS as the normal and negative control, respectively. *n* = 3–4. a) Anti‐A*β* (6E10) immunohistochemistry in the cortex of the animals. Scale bars, 150 µm. b) A*β*‐positive area around cerebral vessels in the hippocampus and cortex. c–g) 10‐month‐old female APP/PS1 mice were treated with RAP‐RL or RL for 4 weeks with the age‐match WT and APP/PS1 mice treated with PBS as the normal and negative control, respectively. In c,d) *n* = 3. In e–g) *n* = 4–5. c) In vivo multiphoton microscopy images of A*β* as indicated by methoxy‐XO4 (green) and cerebral vessels by rhodamine‐dextran (red), Scale bars, 100 µm. d) Quantification of perivascular methoxy‐XO4^+^ A*β* plaques. e–g) ^125^I‐A*β*_1‐42_‐oligomer was injected into the unilateral hippocampus of the animals to evaluate the ability of A*β* clearance. e) Schematic of experimental design. f) Percentage of the degraded ^125^I‐A*β*_1‐42_ oligomer to the total ^125^I‐A*β*_1‐42_ injected into the whole brain. g) Average *γ*‐counter per minute (CPM) in the deep cervical lymph nodes. Data represent mean ± SEM. *P < 0.05, **P < 0.01, ***P < 0.001, ****P < 0.0001, N.S., not significant. g)Student's *t*‐test and b, d, f) one‐way ANOVA with Tukey's multiple‐comparisons test for group comparisons.

Previous work demonstrated that lipoproteins and their mimics possessed the A*β*‐clearance activities.^[^ ^25^ ^]^ To test if RAP‐RL could specifically enhance perivascular A*β* clearance, we administered 13‐month‐old male APP/PS1 mice with RAP‐RL for 4 weeks. Anti‐A*β* (6E10) immunohistochemistry analysis showed that compared with vehicle control (phosphate buffer saline, PBS), RAP‐RL dramatically decreased perivascular A*β* accumulation by 55.24% in the hippocampus and 44.14% in the cortex, respectively (**Figure** **4**a,b). In contrast, RL decreased perivascular A*β* accumulation by 26.00% in the hippocampus and 23.64% in the cortex, respectively (Figure 4a,b). Similar results were also observed in 10‐month‐old female APP/PS1 mice treated with RAP‐RL for 4 weeks (Figure 4c,d). In vivo multiphoton imaging showed that RAP‐RL treatment reduced perivascular A*β* deposition by 41.13%. In contrast, RL reduced perivascular A*β* deposition by 28.13% (Figure 4c,d). Together, these results demonstrated that compared with RL, RAP‐RL more efficiently cleared the perivascular A*β*.

We continued to investigate how RAP‐RL promoted the perivascular A*β* clearance. Although A*β*_1‐40_ and A*β*_1‐42_ are the most common sequential proteolytic products of amyloid *β*‐protein precursor (APP), A*β*_1‐42_ is more toxic than A*β*_1‐40_ due to its greater propensity to aggregate.^[^ ^26^ ^]^ Therefore, we injected ^125^I‐A*β*_1‐42_ oligomer into the unilateral hippocampus (Figure 4e), and utilized trichloroacetic acid (TCA) precipitation to indicate the level of the intact form of A*β*_1‐42_ oligomer.^[^ ^27^ ^]^ Thirty minutes after injection, the ratio of A*β* degradation in the RAP‐RL‐treated mice was 31.19 ± 0.83%, comparable with that in the wild type (WT) animals (29.51 ± 2.08%), and significantly higher than that in the RL‐treated mice (25.02 ± 0.91%), and PBS‐treated controls (21.32 ± 1.70%) (Figure 4f). Moreover, RAP‐RL treatment also dramatically enhanced the clearance of the intrahippocampus injected ^125^I‐A*β*_1‐42_ into the deep cervical lymph nodes (increased by 90.59%) compared with the vehicle control (Figure 4g), indicating that RAP‐RL treatment also accelerates the clearance of A*β* through the lymphatic route.^[^ ^28^ ^]^ In contrast, RL treatment showed no significant difference in the lymphatic A*β* clearance versus vehicle control.

### 2.5 RAP‐RL Normalizes Cerebral Microvasculature and Increases CBF in APP/PS1 Mice

**Figure 5:**
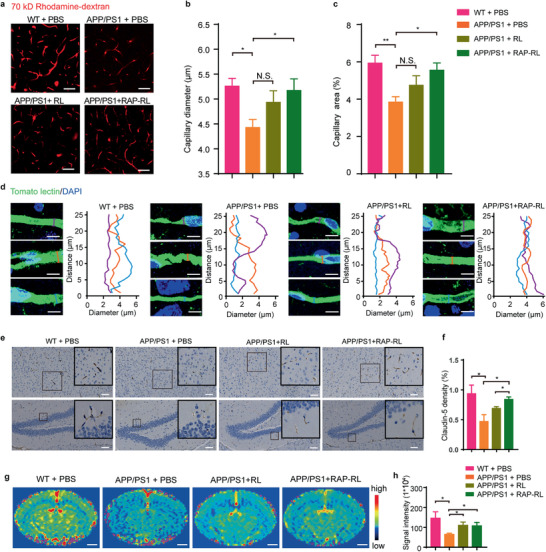
RAP‐RL normalizes the cerebral microvasculature and increases cerebral perfusion in APP/PS1 mice. a–c,g,h) 10‐month‐old female APP/PS1 mice were treated with RAP‐RL or RL for 4 weeks with the age‐match WT and APP/PS1 mice treated with PBS as the normal and negative control, respectively. *n* = 4–5. a) Multiphoton images (Scale bars, 60 µm) of cerebral capillary indicated by rhodamine‐dextran (red) in the cortex. b) Quantification of cerebral capillary diameter and c) capillary area ratio in the cortex. d–f) 13‐month‐old male APP/PS1 mice were treated with RAP‐RL for four weeks with the age‐match WT and APP/PS1 mice treated with PBS as the normal and negative control, respectively. *n* = 3 per group. d) The morphology and radial diameter variation of the cerebral microvasculature as characterized by tomato lectin. Scale bars, 5 µm. e) Immunohistochemical analysis of claudin‐5 in the cortex and hippocampus. Scale bars, 100 µm. f) Quantification of the claudin‐5 in the whole brain. g) Typical CBF maps (Scale bars, 1 mm). h) Quantification of CBF. Data represent the mean ± SEM. *P < 0.05, **P < 0.01, h) Student's *t*‐test and b,c,f) one‐way ANOVA with Tukey's multiple‐comparisons test for group comparisons. N.S., not significant.

Previous work found that the accumulation of perivascular A*β* impaired the integrity of cerebral vessel structure and reduced CBF.^[^ ^29^ ^]^ We also observed capillaries with a smaller diameter and twisted morphology in the brain slices from AD patients (Figure S6a,b, Supporting Information).^[^ ^30^ ^]^ Thus, we sought to explore whether the RAP‐RL‐induced clearance of perivascular A*β* could improve the structure and function of cerebral vessels. Consistent with that found in the brain of AD patients, those APP/PS1 mice showed aberrant vascular network with the capillary diameter (4.43 ± 0.16 µm) and capillary density (3.84 ± 0.29%) in the cortex significantly lower than those in the age‐matched WT mice (5.27 ± 0.16 µm and 5.92 ± 0.42%, respectively) (**Figure** **5**a–c). Strikingly, four‐week RAP‐RL treatment largely reversed these changes (vessel diameter: 5.20 ± 0.25 µm, vessel density: 5.55 ± 0.39%) (Figure 5b,c). The same effect was observed in 13‐month‐old male APP/PS1 mice, four‐week RAP‐RL treatment efficiently improve the cortical microvasculature, making them larger, more orderly and smoother (Figure 5d). To further validate the effects of RAP‐RL on vascular normalization, we continued to determine the effect of RAP‐RL on the expression of the BBB tight‐junction proteins including claudin‐5 and ZO‐1, which were significantly altered under AD.^[^ ^31^ ^]^ Consistent with that found in bEnd.3 cells, in 13‐month‐old male APP/PS1 mice, RAP‐RL significantly increased claudin‐5 density (by 78.24%) comparing with the vehicle control (Figure 5e,f). Such an effect was also significantly stronger than RL (increased by 46.31% compared with the vehicle control). Collectively, these results implicated that RAP‐RL can largely recover the structure and the function of BBB.

CBF homeostasis is essential for normal brain function, and reduced CBF is frequently found in AD patients and animal models.^[^ ^12^ ^]^ As RAP‐RL could normalize the cerebrovasculature, we further evaluated its effect on cerebral blood perfusion. Compared with that in the WT littermates, CBF in the APP/PS1 mice decreased by 50.36%, but was fully restored upon the treatment with RAP‐RL as indicated by contrast perfusion‐enhanced magnetic resonance imaging (MRI) (Figure 5g,h). These results demonstrated that RAP‐RL could normalize cerebrovasculature and restore cerebral perfusion.

### 2.6 RAP‐RL Normalizes NVU Structure and Improves NVU Response

**Figure 6:**
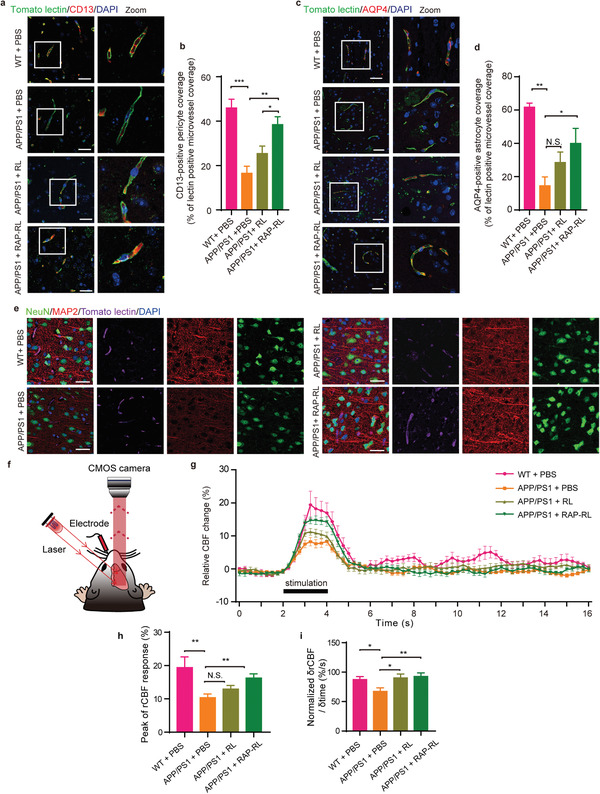
RAP‐RL normalizes the NVU structure and improves NVU response. a–e) 13‐month‐old male APP/PS1 mice were treated with RAP‐RL or RL for 4 weeks with the age‐matched WT and APP/PS1 mice treated with PBS as the normal and negative control, respectively. *n* = 3–4. a) Immunofluorescence of CD13 and tomato lectin in the cortex. Scale bars, 20 µm. b) Quantification of pericyte (CD13) coverage. c) Immunofluorescence of AQP4 and tomato lectin in the cortex. Scale bars, 40 µm. d) Quantification of AQP4 coverage. e) Immunofluorescence of NeuN (neuron), MAP2 (dendrite), and tomato lectin costaining in the cortex. Scale bars, 20 µm. f–i) 10‐month‐old female APP/PS1 mice were treated with RAP‐RL and RL for 4 weeks with the age‐match WT and APP/PS1 mice treated with PBS as the normal and negative control, respectively. *n* = 6–8. f) Schematic of experimental design. g) Time courses of relative CBF changes in the barrel cortex, in response to an electrical whisker pad stimulus. h) Quantification of the peak relative CBF changes in the cortex, two measurements were taken from each mouse. i) Quantification of the slope of CBF response. Data represent the mean ± SEM. *P < 0.05, **P < 0.01, ***P < 0.001, N.S., not significant. One‐way ANOVA with Tukey's multiple‐comparisons test for group comparisons.

Distinct from the peripheral vasculature, the cerebral vessels have been appreciated to function as a multicell‐associated vascular system termed as NVU, which involves endothelial cells, mural cells, astrocytes, microglia, and neurons. We then interrogated whether RAP‐RL processes the ability to restore the structure and function of NVU. Pericytes, a type of mural cells of the capillary vessel wall, play a critical role in the stabilization of the capillary wall and the maintenance of the BBB.^[^ ^32^ ^]^ By immunostaining pericytes with the marker CD13, we found that, similar with that in the WT mice (45.63 ± 2.05%), the ratio of CD13^+^ cells colocalized with tomato lectin‐positive endothelial cells in the RAP‐RL‐treated mice was 37.61 ± 4.57%, increased by 134.77% (**Figure** **6**a,b) compared with that in the PBS‐treated controls (16.02 ± 2.62%). RL treatment (26.66 ± 2.64%) also increased the colocalization between CD13 and endothelial cells, but in a much less extent (Figure 6a,b). Likewise, following the treatment with RAP‐RL, *α*‐SMA, another marker of mural cells (including both pericytes and smooth muscle cells, SMCs), regained its expression and nicely coated on the cerebral vessels (Figure S7, Supporting Information). Moreover, such restored CD13 and *α*‐SMA expression well coincided with the normalized vascular morphology, suggesting the improved coupling between pericytes/SMCs and the endothelial cells following the RAP‐RL treatment.

In the NVU, astrocytes are well positioned with feet to transmit neural signaling to microvasculature.^[^ ^33^ ^]^ Astroglial water channel aquaporin‐4 (AQP4), which localizes at the perivascular astrocytic end feet ensheathing the cerebral vasculature, mediates brain waste clearance through the lymphatic pathway.^[^ ^34^ ^]^ The expression of AQP4 along the cerebral vasculature of APP/PS1 mice was enhanced by 179.37% following the RAP‐RL treatment compared with that in the PBS‐treated controls. RL treatment also increased the colocalization between AQP4 and endothelial cells, but to a much less extent (increased by 96.56%) (Figure 6c,d). Astrogliosis is another hallmark of AD pathogenesis.^[^ ^35^ ^]^ We then determined the level of astrogliosis through glial fibrillary acidic protein (GFAP) immunostaining, finding that the expression of GFAP in the cortex of APP/PS1 mice was 23.33 ± 3.54%, decreased by 68.37% (7.38 ± 0.30%) following RAP‐RL treatment (Figure S8, Supporting Information).

Microglia is another kind of glial cell in the NVU, responding to changes in BBB and neuronal activity.^[^ ^36^ ^]^ RAP‐RL also induced a remarkable response in microglia as indicated by the increased Iba1 immunoreactivity. Microglia exhibited an enlarged cell body (increased by 27.43%) and tended to cluster following the RAP‐RL treatment (Figure S9a, Supporting Information). Given that the extracellular A*β* is mainly phagocytosed and cleared by microglia in the brain, we coimmunostained A*β* (6E10) and microglia (Iba1), finding that RAP‐RL treatment significantly enhanced the colocation between A*β* aggregates and microglia (Figure S9b,c, Supporting Information), and increased the number of microglia surrounding A*β* (Figure S9b,d, Supporting Information). Interestingly, such enhanced microglial recruitment did not induce neuroinflammation. On the contrary, the levels of proinflammatory cytokines including TNF‐*α* and IL‐6 in the hippocampus and cortex significantly decreased (TNF‐*α*: reduced by 40.88% in the hippocampus and 45.28% in the cortex, respectively; IL‐6: reduced by 6.82% in the hippocampus and 28.58% in the cortex, respectively) following the RAP‐RL treatment (Figure S10, Supporting Information). These data suggested that RAP‐RL markedly enhanced the recruitment of microglia surrounding A*β* plaques without aggravating neuroinflammation.

Neuron, a core cell type in the NVU, is directly relevant to learning and memory.^[^ ^9^ ^]^ The recoupling between neuron and cerebrovasculature is an important hallmark of the reconstruction of NVU.^[^ ^9^ ^]^ Compared with the WT mice, APP/PS1 mice exhibited shrinking neuron soma, loss of dendrites, and uncoupling between neurons and cerebral microvasculature (Figure 6e). Following the RAP‐RL treatment, although the number of NeuN^+^ neuron was only slightly increased, the expression levels of NeuN (10.93 ± 0.87%, indicates neuron soma) and MAP2 (20.64 ± 2.98%, indicates dendrites) were both significantly elevated (by 27.5% and 49.12%, respectively) compared with that in the PBS‐treated controls, and similar with that in WT mice (9.72 ± 0.36% and 19.01 ± 2.64%, respectively) (Figure S11, Supporting Information). Such recovery of neuron and NVU morphology is associated with the improvement of neurovascular coupling as indicated by a whisker pad electrical stimulation and laser speckle contrast imaging (LSCI) experiment (Figure 6f).^[^ ^37^ ^]^ The peak CBF response indicates neurovascular response. As illustrated by LSCI time courses, similar with that in the WT mice (19.39 ± 3.21%), the average peak CBF response in RAP‐RL group was 16.25 ± 1.28%, increased by 56.70% and 32.02% compared with that in the PBS‐treated controls (10.37 ± 1.09%) and RL‐treated group (12.93 ± 1.12%), respectively (Figure 6g,h). Specifically, the slope of CBF response following RAP‐RL treatment was significantly higher than that in the vehicle control, indicating higher response efficiency of NVU (Figure 6i). Together, these results suggested that RAP‐RL could normalize the NVU structure and improve the NVU response.

### 2.7 RAP‐RL Improves Memory Function in APP/PS1 Mice

**Figure 7:**
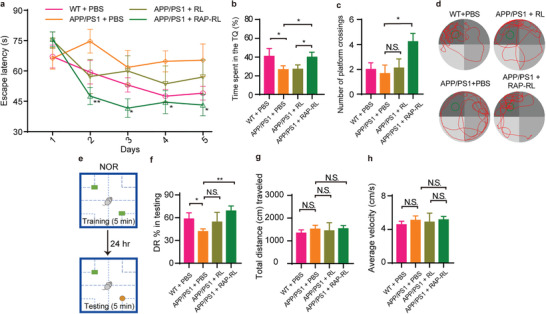
RAP‐RL improves cognitive function in APP/PS1 mice. a–h) 10‐month‐old female APP/PS1 mice were treated with RAP‐RL or RL for 4 weeks with the age‐match WT and APP/PS1 mice treated with PBS as the normal and negative control, respectively. *n* = 8–9. a) Escape latency of APP/PS1 mice in the MWM test. b) Time spent in target quadrant (TQ), c) the number of platform crossings and d) representative track path of mice during the probe trial. e) Schematic of NOR experimental design. f) Discrimination ratio (DR) in NOR test. g) Total distance (cm) and h) average velocity (cm s^−1^) during novel object recognition test. Data represent the mean ± SEM. *P < 0.05, **P < 0.01 and ***P < 0.001, b) Student's *t*‐test, a) two‐way or c,f–h) one‐way ANOVA with Tukey's multiple comparisons test. N.S., not significant.

Morris water maze (MWM) test was performed to evaluate the spatial memory of the animals. Compared with WT mice, PBS‐treated APP/PS1 mice showed significant defects in spatial learning and memory function. Treatment with RAP‐RL significantly decreased escape latency in the training trials (**Figure** **7**a). During the probe trial, those mice received RAP‐RL spent a significantly longer time exploring the target quadrant (TQ) (Figure 7b,d) and achieved a higher number of platform crossing (Figure 7c), versus the RL‐treated and PBS‐treated APP/PS1 mice. In addition, the alleviation of these phenotypes upon RAP‐RL treatment was not due to the change of swimming speed (Figure S12, Supporting Information). These data indicated that RAP‐RL efficiently improves spatial learning and memory function in APP/PS1 mice.

Novel object recognition (NOR) test, another commonly used behavioral assay, was also applied to evaluate the learning and memory ability of the AD model mice (Figure 7e). On the training day, the animals from the different groups did no exhibit preference for the objects (Figure S13, Supporting Information). On the testing day, compared with the PBS‐treated controls, RAP‐RL‐treated mice spent a significantly longer time examining the novel object than the familiar object with a higher discrimination ratio (DR) (68.95 ± 6.51% in RAP‐RL‐treated group vs 41.80 ± 3.46% in PBS‐treated controls) (Figure 7f). In contrast, slightly but not significant improvement in DR was achieved in those RL‐treated animals (54.36 ± 12.60%). Importantly, the various groups of animals did not differ significantly in the travel distance (Figure 7g) and average velocity (Figure 7h) during the test, indicating that the different recognition index was not due to their different activity.

In addition, to evaluate the safety of RAP‐RL, we also determined the body weight, renal function and liver function of those mice after the 4‐week treatment of RAP‐RL. No difference in weight change was observed among the various treatment groups (Figure S14, Supporting Information). Serum biochemistry analysis and hematoxylin‐eosin (H&E) staining of major organs also found no substantial changes in renal function and liver injury markers such as blood urea nitrogen (BUN), creatinine (Cr), uric acid (UA), alanine aminotransferase (ALT), aspartate aminotransferase (AST), AST:ALT, and alkaline phosphatase (ALP) in the RAP‐RL‐treated mice (Figure S15, Supporting Information). Notably, RAP‐RL restored triglyceride levels to normal levels, comparable to that in the WT mice (Figure S15e, Supporting Information). These results strongly demonstrated that RAP‐RL is highly biocompatible.

## 3 Discussion

Increasing evidences support that cerebrovascular dysfunction is critical to the pathogenesis of AD and contributes substantially to the permanent loss of neuronal function. However, due to the sophisticated nature of NVU and the cerebrovascular network, current therapeutics for the rescue of cerebrovascular function failed to alleviate the cognitive deficits in AD model mice or AD patients.^[^ ^7a,b^ ^]^ Accordingly, we proposed that multifunctional therapeutics which can target the damaged brain blood vessels, remediate the multicomponents of NVU and thus normalize the cerebrovascular system would provide a promising alternative to AD treatment. To test this hypothesis, here we developed a multifunctional biomimetic nanostructure RAP‐RL that composed of RAP, GM1, and ApoE3 to target and normalize the cerebral neurovascular system. As a result, RAP‐RL exhibited strong efficacy in recovering the function of individual cellular participants in NVU and improving the hippocampal‐dependent recognition and spatial memory activity in AD.

One of the key properties of RAP‐RL is its relatively specific accumulation along the cerebral microvasculature (Figure 2). In contrast, RL, the control formulation without RAP, did not show specific association with the vascular walls, but enter the parenchyma evenly. The major reason for the accumulation of RAP‐RL along the cerebral microvasculature lies in that a key component of the nanostructure, RAP, a peptide derived from RAGE's nature ligand S100P, can specifically bind to RAGE overexpressing on the endothelium of AD cerebral vasculature.^[^ ^15^, ^38^ ^]^ Notably, we also evaluated the interaction between RAP‐RL and cerebral blood vessels in brain sections from AD patients, observing the accumulation of RAP‐RL along the vessel walls (Figure S3, Supporting Information). With regard to RAGE targeting, Azeliragon is an orally bioavailable small molecule inhibitor of RAGE, which has been tested for the treatment of mild AD (NCT02080364). Unfortunately, Azeliragon was terminated in phase III clinical trials as it failed to show any benefit over placebo on cognitive endpoints in patients with probable AD.^[^ ^39^ ^]^ Differently, RAP‐RL is a novel multifunctional lipoprotein‐like nanostructure, which can alleviate the various pathological changes of NVU. Moreover, RAP‐RL is a biomimetic nanostructure characterized with long circulation time in the blood and biocompatible. These properties endow RAP‐RL great potential in clinical application for cerebrovascular remodeling.

Interestingly, compared with RL which can cross the BBB and evenly distributed in the parenchyma, RAP‐RL which majorly accumulated along the walls of microvasculature, more powerfully reduced perivascular A*β* deposition (Figure 4). RAP‐RL‐associated reduction of perivascular A*β* accumulation could be caused by at least three reasons. First, the binding of RAP‐RL to RAGE may competitively inhibit RAGE‐mediated A*β* transport into the brain, similar with the RAGE inhibiter Azeliragon.^[^ ^40^ ^]^ Secondly, ApoE3 and GM1, the two components of RAP‐RL, possess a high binding affinity to A*β* to promote its intracerebral degradation and efflux across BBB.^[^ ^25^, ^41^ ^]^ Third, the lymphatic system, which is impaired in AD with the reduction of AQP4 on astrocytic end feet,^[^ ^34^ ^]^ largely recovered due to the RAP‐RL‐induced astrocytes normalization (Figure 6c,d, Figure S8, Supporting Information) could also enhance A*β* clearance through the lymphatic system (Figure 4g). Collectively, following the RAP‐RL treatment, the reduced entrance and enhanced clearance of A*β* could both contribute to the significantly decreased perivascular A*β* depositions in the brain of APP/PS1 mice.

Altered morphology of cerebral vasculature and reduced CBF are critical pathological changes in AD.^[^ ^3^, ^30^ ^]^ In human AD brain tissues, compared with that in the control brains, we also witnessed more capillaries with small diameter and twisted morphology as reported previously (Figure S6, Supporting Information). Exhilaratingly, we found that RAP‐RL treatment largely restored the morphology of the cerebral vasculature and efficiently increased CBF in AD model mice (Figure 5). Perivascular A*β* impairs the cerebral vessels structure and CBF.^[^ ^22^, ^29^ ^]^ Therefore, the RAP‐RL‐induced diminished peripheral A*β* deposit would definitely contribute to the improved brain vasculature morphology and increased CBF. In addition, a key component of RAP‐RL, ApoE3, has been shown to regulate pericyte‐mediated cerebrovascular integrity and anti‐inflammatory reaction.^[^ ^18^, ^19^ ^]^ After the RAP‐RL treatment, normalized CD13 and *α*‐SMA expression were found well coincided with the normalized vascular morphology, indicating that RAP‐RL improved the coupling between pericytes/SMCs and the endothelial cells (Figure 6a,b, Figure S7, Supporting Information). It has been well documented that pericytes play an important role in stabilizing the vessel wall and thus regulate the brain blood perfusion.^[^ ^19^, ^29^ ^]^ Therefore, the reduced perivascular A*β* accumulation together with the improved cross‐talk between pericytes and endothelial cells could both contribute to the RAP‐RL‐mediated normalization of the structure and function of the cerebrovascular system. Moreover, as inflammatory factors including IL‐1, IL‐6, GM‐CSF, IL‐12 and IL‐23, and TNF‐*α* were all upregulated under AD condition.^[^ ^23^ ^]^ RAP‐RL, which alleviates neuroinflammation (Figure S10, Supporting Information) might also protect the noninjured cerebral microvessels from the neuroinflammatory injury. RL, the control formulation, which can also reduce perivascular A*β* deposition and regulate pericyte‐mediated cerebrovascular integrity, also partially restored the morphology of the cerebral vasculature but to a less extent (Figures 4 and 5). Collectively, these findings highlight the importance of the microvasculature‐targeting properties of RAP‐RL for restoring the structure and function of the cerebrovascular system.

Besides the general improved cerebrovasculature morphology and improved cross‐talk between pericytes and endothelial cells, restoration of other multiple components of NVU also occurred following the RAP‐RL treatment (Figure 6, Figures S7–S11, Supporting Information). In the NVU, astrocytes act as a “bridge,” sensing and delivering synaptic activity to cerebrovasculature through multiple end‐feet that cover the cerebral microvessels.^[^ ^9^ ^]^ In AD, reactive astrogliosis is upregulated corresponds to the severity of astroglial activation and high levels of proinflammatory cytokines and the hypertrophic processes make aberrant contacts with nearby cerebral vessels and neurons, leading to neurovascular uncoupling.^[^ ^42^ ^]^ Moreover, the expression of AQP4 on astrocytic endfeet decrease and their perivascular polarization is inhibited, ultimately leading to lymphatic clearance dysfunction.^[^ ^11^, ^43^ ^]^ Here, we found that RAP‐RL, more efficiently than RL, decreased the level of astrogliosis, suggested that the inflammatory environment in the brain is remarkably alleviated (Figure S8, Supporting Information). At the same time, RAP‐RL upregulated expression of AQP4 along the cerebrovasculature (Figure 6c,d) and improved clearance of the intrahippocampus‐injected ^125^I‐A*β*_1‐42_ into the deep cervical lymph nodes (Figure 4g).

Microglia is another type of glia in the NVU, responding to BBB damage and neuron injury.^[^ ^36^, ^44^ ^]^ In this study, we found that RAP‐RL induced morphological alteration and A*β* phagocytic responses in microglia. Interestingly, the enhanced microglial phagocytic activity did not increase neuroinflammation. In contrast, declined levels of proinflammatory mediators such as TNF‐*α* and IL‐6 was observed after the RAP‐RL treatment (Figure S10, Supporting Information). ApoE3, an effective anti‐inflammatory molecule,^[^ ^18^ ^]^ could largely contribute to the anti‐inflammatory effect. In addition, those RAP‐RL crossing the BBB might directly modulate the immune‐phenotype of microglia. Further study is needed to explore whether the effects of RAP‐RL on microglia are synergistic or simply addition of the individual effects.

Neurons play a central role in learning and memory. As a key component of RAP‐RL, GM1 processes neuroprotective activity. But its in vivo application is largely limited by the negligible amount of GM1 that can cross the BBB to access its target.^[^ ^13^ ^]^ To circumvent this obstacle, here, we incorporated GM1 into the reconstituted lipoprotein to realize its delivery to the injured NVU. As a result, RAP‐RL powerfully reversed the shrinkage of neuron soma and loss of dendrites and restored neurovascular coupling (Figure 6). It is also very likely that RAP‐RL normalized cerebral vessels, regained pericytes covering, increased CBF, reduced neuroinflammation, all of which ultimately rendered neurons normally functional.^[^ ^6a^ ^]^

To evaluate the effect of RAP‐RL on cognition deficits, here we applied APP/PS1 mice, which exhibited apparent dysfunction of learning and memory from 6 to 8 months,^[^ ^45^ ^]^ as the animal model. It was found that in 10‐month‐old female APP/PS1 mice, compared with the noncerebral vasculature targeting formulation RL, RAP‐RL more efficiently rescued the cognitive deficits of AD model mice at a very low dose (0.34 mg kg^−1^ DMPC). Such effect is also more efficient than our previously reported ApoE3‐incorporated reconstituted high density lipoprotein (ApoE3‐rHDL), composed of only DMPC and ApoE3, which can cross the BBB, capture A*β* and facilitate its degradation by glial cells, efficiently rescue the memory loss in senescence‐accelerated prone mouse (SAMP8) model at the dose of 5 mg kg^−1^.^[^ ^25^ ^]^ BBB is a crucial obstacle for the delivery of AD‐modifying agents.^[^ ^46^ ^]^ ApoE3‐rHDL and RL need to cross the BBB to facilitate A*β* clearance. In contrast, as designed, RAP‐RL only needs to bind to the endothelial cells to remediate the multicomponents of NVU. This could be the major reason that RAP‐RL achieved significant cognitive improvement at a much lower dose. Moreover, the safety of RAP‐RL is not only demonstrated by its biomimetic materials components, but also validated by the in vivo tests. No obvious side effect and toxicity was observed following the RAP‐RL treatment as evidenced by body weight, hematoxylin‐eosin (H&E) staining of major organs and serological analysis (Figure S15, Supporting Information). The effectiveness and safety of RAP‐RL suggest a good clinical application prospect.

In addition to improving drug development, the nanostructure designed here might also enable new research into the mechanisms linking cerebrovascular dysfunction and the progression of various dementias including AD. Our findings demonstrate NVU as a potential target of AD treatment and support the hypothesis that normalizing cerebrovascular network could restore the global ability of the NVU and ameliorate multicellular function in AD. Deeper investigations of a potential mechanistic link between cerebrovascular network dysfunction and AD progression are warranted.

## 4 Conclusion

In summary, we have developed the multifunctional nanostructure RAP‐RL, which efficiently targeted the injury cerebral microvasculature, mitigated inflammation, reduced perivascular A*β*, and protected cerebrovasculature. Through the synergistic effect of RAP‐RL, the integrity and function of NVU were regained and the cognition was improved in AD model mice. We provide compelling evidence that multifunctional therapeutics which can target the damaged brain blood vessels, remediate the multicomponents of NVU and thus normalize the cerebrovascular system would provide a promising alternative to AD treatment.

## Conflict of Interest

The authors declare no conflict of interest.

## Supporting information

Supporting Information
