# Supplementary material for: Multifunctional Nanostructure RAP‐RL Rescues Alzheimer's Cognitive Deficits through Remodeling the Neurovascular Unit
Source: Adv Sci (Weinh). 2020 Dec 10;8(2):2001918. doi: 10.1002/advs.202001918 (PMC7816710; doi:10.1002/advs.202001918)
Supplement: Supplementary file 1 — Supporting Information [file ADVS-8-2001918-s001.pdf]

WILEY-VCH

© 2020 Wiley-VCH GmbH

ADVANCED SCIENCE Open Access

Supporting Information

for *Adv. Sci.*, DOI: 10.1002/advs.202001918

Multifunctional Nanostructure RAP-RL Rescues Alzheimer's Cognitive Deficits through Remodeling the Neurovascular Unit

Qian Zhang, Qingxiang Song, Xiao Gu, Mengna Zheng, Antian Wang, Gan Jiang, Meng Huang, Huan Chen, Yu Qiu, Bin Bo, Shanbao Tong, Rong Shao, Binyin Li, Gang Wang, Hao Wang, Yongbo Hu, Hongzhuan Chen, Xiaoling Gao**

Multifunctional Nanostructure RAP-RL Rescues Alzheimer's Cognitive Deficits through Remodeling the Neurovascular Unit

Qian Zhang, Qingxiang Song, Xiao Gu, Mengna Zheng, Antian Wang, Gan Jiang, Meng Huang, Huan Chen, Yu Qiu, Bin Bo, Shanbao Tong, Rong Shao, Binyin Li, Gang Wang, Hao Wang, Yongbo Hu, Hongzhuan Chen, Xiaoling Gao**

Dr. Q. Zhang, Q. Song, X. Gu, M. Zheng, A. Wang, M. Huang, H. Chen, G. Jiang, Y. Hu, Prof. Y. Qiu, R. Shao, H. Wang, H. Chen, Prof. X. Gao
Department of Pharmacology and Chemical Biology
State Key Laboratory of Oncogenes and Related Genes
Shanghai Universities Collaborative Innovation Center for Translational Medicine
Shanghai Jiao Tong University School of Medicine
280 South Chongqing Road, Shanghai 200025, China
Email: shellygao1@sjtu.edu.cn; hongzhuan_chen@hotmail.com
Dr. B. Bo, Prof. S. Tong
School of Biomedical Engineering and Med-X Research Institute
Shanghai Jiao Tong University
800 Dongchuan Road, Shanghai 200240, China
Prof. G. Wang, Dr. B. Li
Department of Neurology & Neuroscience Institute
Ruijin Hospital affiliated to Shanghai Jiao Tong University School of Medicine
197 Rui Jin Er Road,Shanghai 200025 China
Prof. H. Chen
Institute of Interdisciplinary Integrative Biomedical Research, Shuguang Hospital
Shanghai University of Traditional Chinese Medicine
1200 Cailun Road, Shanghai 201210, China

Keywords: Alzheimer's disease, multifunctional nanostructure, cerebrovasculature, the NVU remodeling

Supporting Information

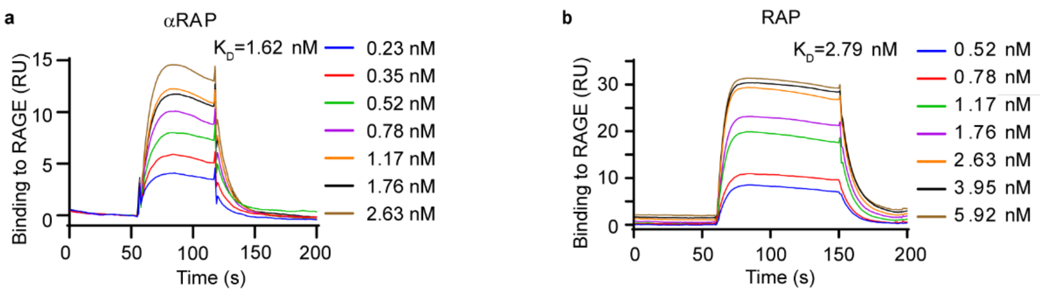

Figure S1. Concentration-dependent binding of RAP or αRAP to RAGE in vitro. a) Binding of αRAP and b) RAP to RAGE protein, evaluated by surface plasmon resonance (SPR) analysis, respectively.

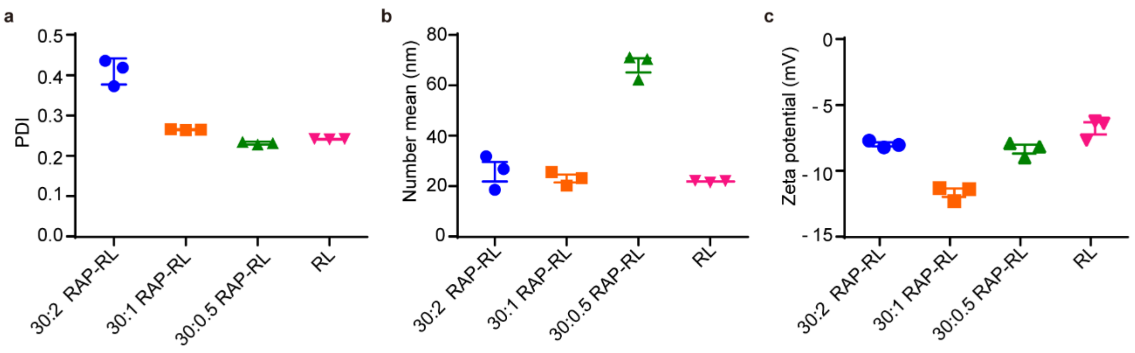

Figure S2. Characterization of RAP-RL with different density of αRAP peptide. a) Polymer dispersion index (PDI), b) Particle size and c) Zeta potential of RAP-RL composed with αRAP and DMPC liposomes at different ratios of 30:2, 30:1 and 30:0.5 (αRAP: DMPC, M/M, n=3).

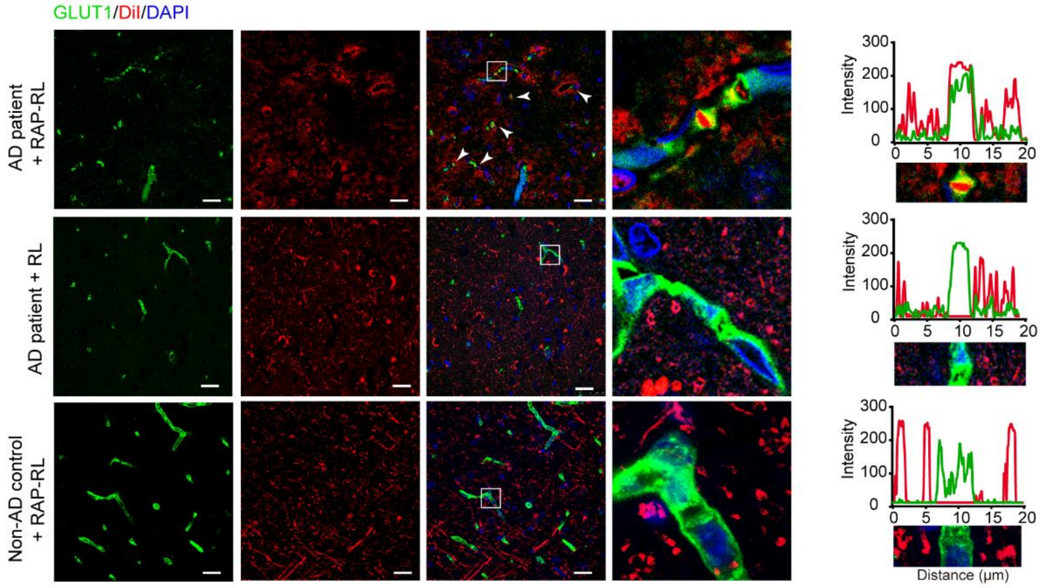

Figure S3. RAP-RL efficiently binds to cerebral vasculature from AD patients. The cortical slices were incubated with DiI-RAP-RL (containing 10 μg/mL DMPC) for 3.5 h. DiI-RAP-RL (red) was found to bind to cerebral vasculature (indicated by GLUT1 immunofluorescent staining, green) from AD patients. Arrowheads represent RAP-RL bind to cerebral vasculature Scale. bars, 25 μm.

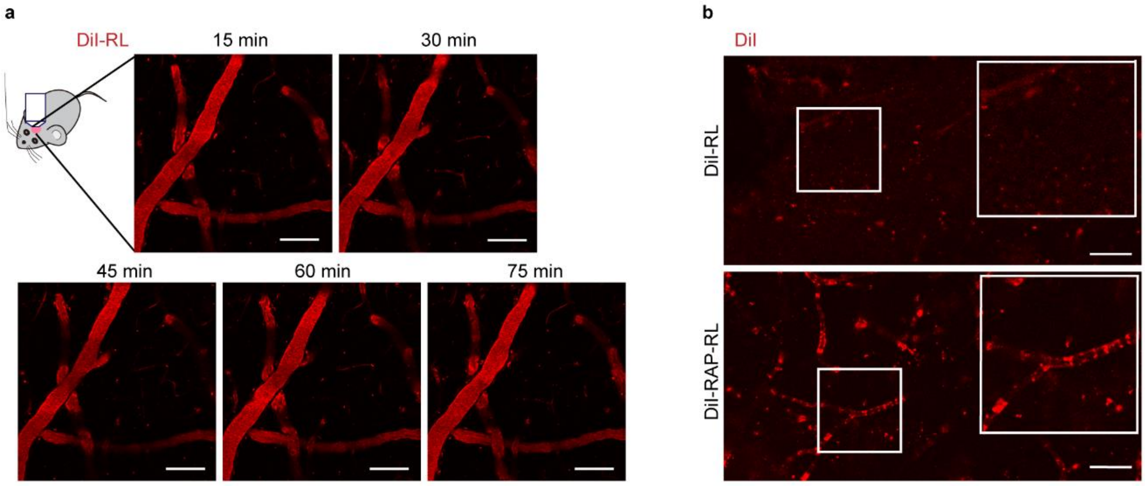

Figure S4. RAP-RL but not RL binds to cerebral vessels effectively. a) DiI-RL did not accumulate along the cerebrovasculature over time under multi-photon microscopy. Scale bar, 100 μm. b) RAP-RL was found majorly accumulated along the cerebrovasculature. At 3.5 h

after administration, the APP/PS1 mice were perfused, and the whole brains were harvested and subjected to multi-photon microscopy analysis. Scale bars, 50 µm.

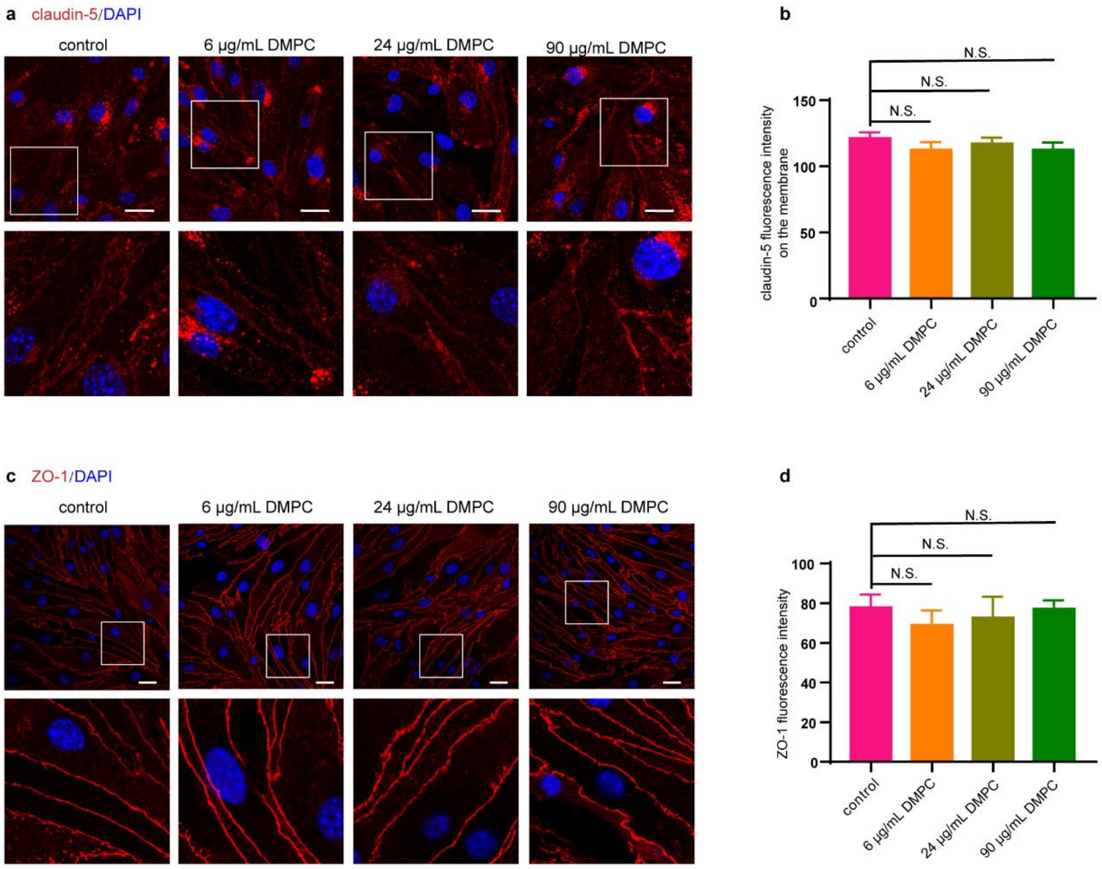

Figure S5. In the absence of A$\beta_{1\text{-}42}$ oligomer, RAP-RL did not change claudin-5 and ZO-1 expression in bEnd.3. a) Claudin-5 immunofluorescence (red) in bEnd.3 cells following RAP-RL treatments containg 6 to 90 µg/mL DMPC. DAPI (blue) was used to stain nuclei. Scale bar, 20 µm. b) Quantification of claudin-5 fluorescence intensity on the cell membrane (n=4). c) ZO-1 immunofluorescence (red) bEnd.3 cells following RAP-RL treatments containing 6 to 90 µg/mL DMPC. DAPI (blue) was used to stain nuclei. Scale bar, 20 µm. d) Quantification of ZO-1 fluorescence intensity (n=4). N.S., not significant. One-way ANOVA with Tukey's multiple-comparisons test for group comparisons.

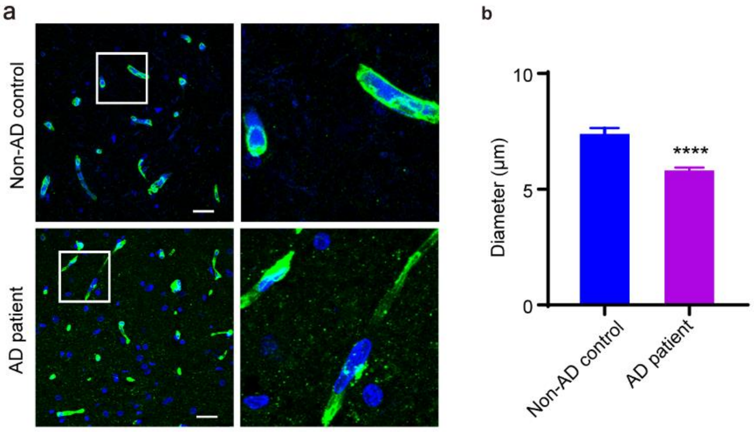

Figure S6. Capillaries with smaller diameter and twisted morphology observed in the brain of AD patients. a) Typical morphology of capillary in human cortical slices from AD patients and non-AD controls as indicated by GLUT1 (green). DAPI labeling cell nuclei. Scale bars, 25 μm. b) Quantification of the diameter of cerebral capillary in AD patients and non-AD controls (n=3, 21 ROI per group). Data are mean ± SEM. ****P <0.0001, Student's t-test for group comparisons.

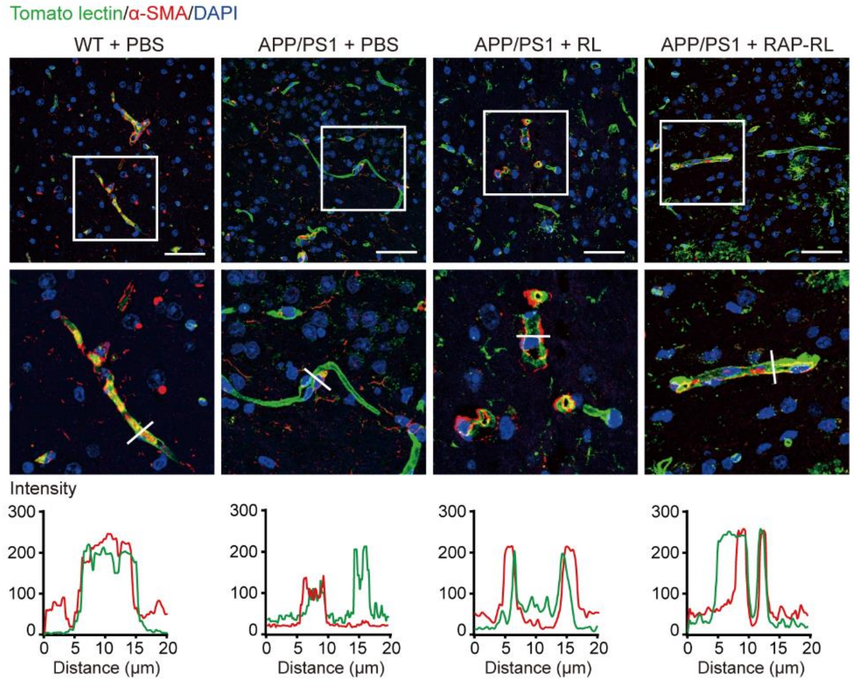

Figure S7. RAP-RL reverses the expression of α-SMA. Thirteen-month-old male APP/PS1 mice were treated with RAP-RL or RL for four weeks with the age-match WT and APP/PS1 mice treated with PBS as the normal and negative control, respectively. Immunofluorescence

of α-SMA and tomato lectin in the cortex. DAPI labels cell nuclei. Scale bars, 20 μm. Line intensity profiles of α-SMA (red line) and tomato lectin (green line) staining.

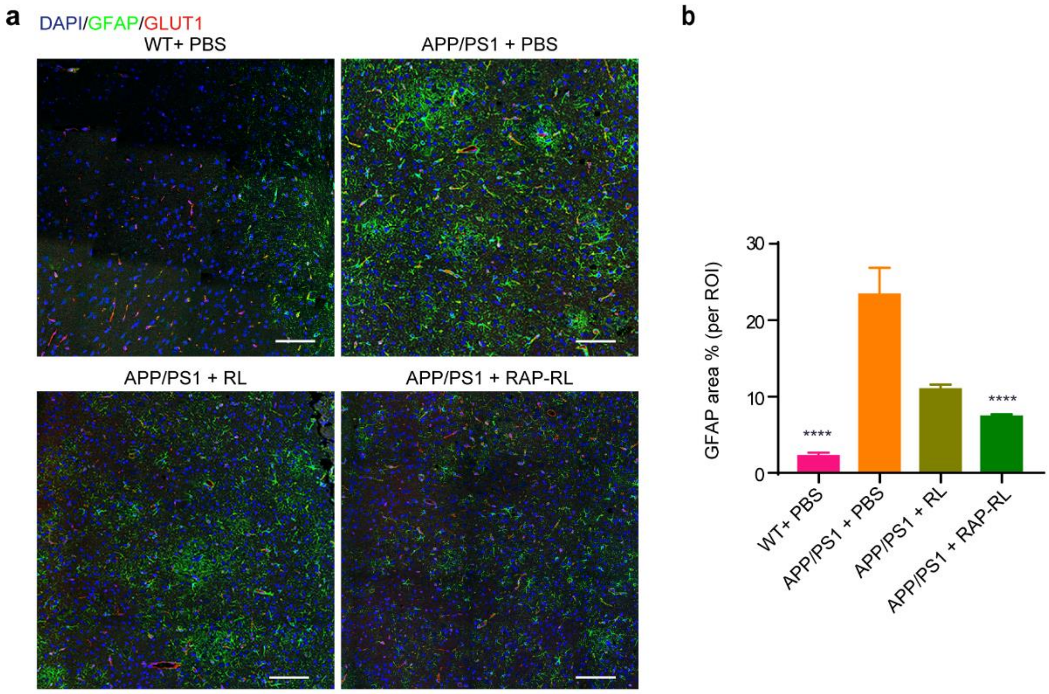

Figure S8. RAP-RL reduces astrogliosis. Thirteen-month-old male APP/PS1 mice were treated with RAP-RL or RL for four weeks with the age-match WT and APP/PS1 mice treated with PBS as the normal and negative control, respectively. n=3-4. a) Immunofluorescence of GFAP and GLUT1 in the cortex. DAPI labels cell nuclei, and GLUT1 labels cerebral vessels. Scale bars, 100 μm. b) Quantification of $GFAP^{+}$ area density following the different treatments. (9-12 ROI from per group). Data represent the mean ± SEM. ***P <0.001, ****P <0.0001. One-way ANOVA with Tukey's multiple-comparisons test for group comparisons.

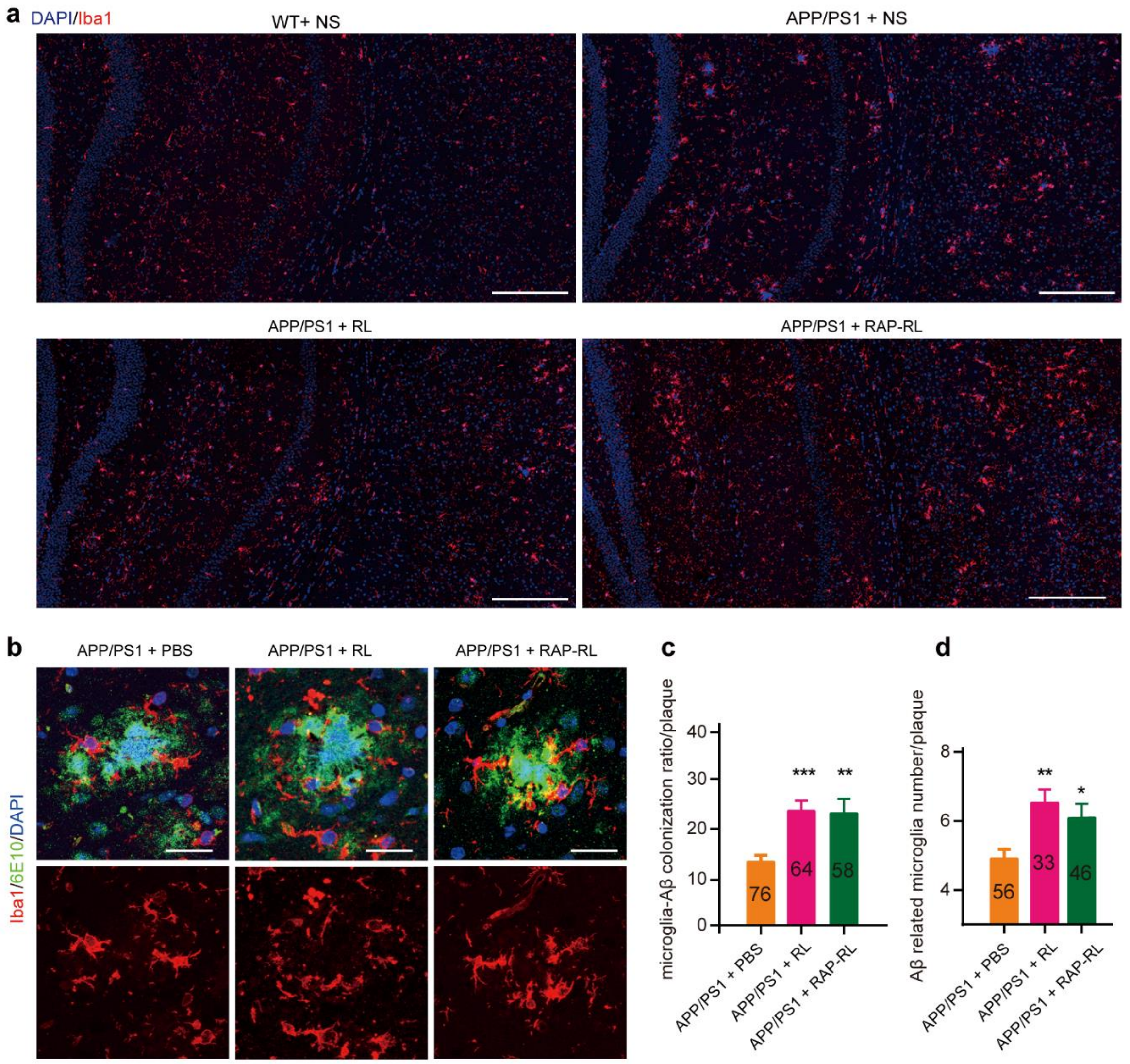

Figure S9. RAP-RL enhances microglial recruitment around the Aβ plaques and promotes Aβ phagocytosis. Ten-month-old female APP/PS1 mice were treated with RAP-RL or RL for four weeks with the age-match WT and APP/PS1 mice treated with PBS as the normal and negative control, respectively. n=3-4. a) Immunofluorescence of Iba1 in the cortex and hippocampus, DAPI labels cell nuclei. Scale bars, 250 μm. b) Immunofluorescent staining with anti-Iba1 and anti-Aβ (6E10) antibodies in the cortex of APP/PS1 mice. Scale bars, 25 μm. c) Percentage area of Iba1-Aβ co-localization. d) Number of Iba1-positive microglia around the Aβ plaques. Number of ROIs analyzed shown on bars. Data represent the mean ± SEM. **P* <0.05, ***P* <0.01, ****P* <0.001. One-way ANOVA with Tukey's multiple-comparisons test for group comparisons. N.S., not significant.

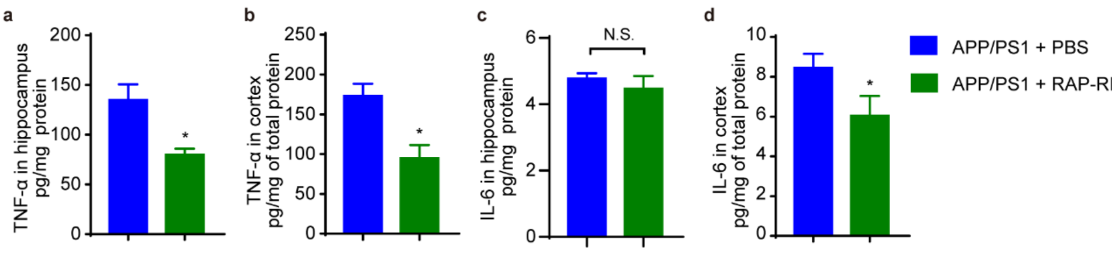

Figure S10. RAP-RL alleviates neuroinflammation in APP/PS1 mice. Ten-month-old female APP/PS1 mice were treated with RAP-RL or PBS for four weeks. n=3-4. a) Levels of TNF-α in the hippocampus and b) cortex. c) Levels of IL-6 in the hippocampus and d) cortex. Data represent the mean ± SEM. *P <0.05. Student's t-test for group comparisons. N.S., not significant.

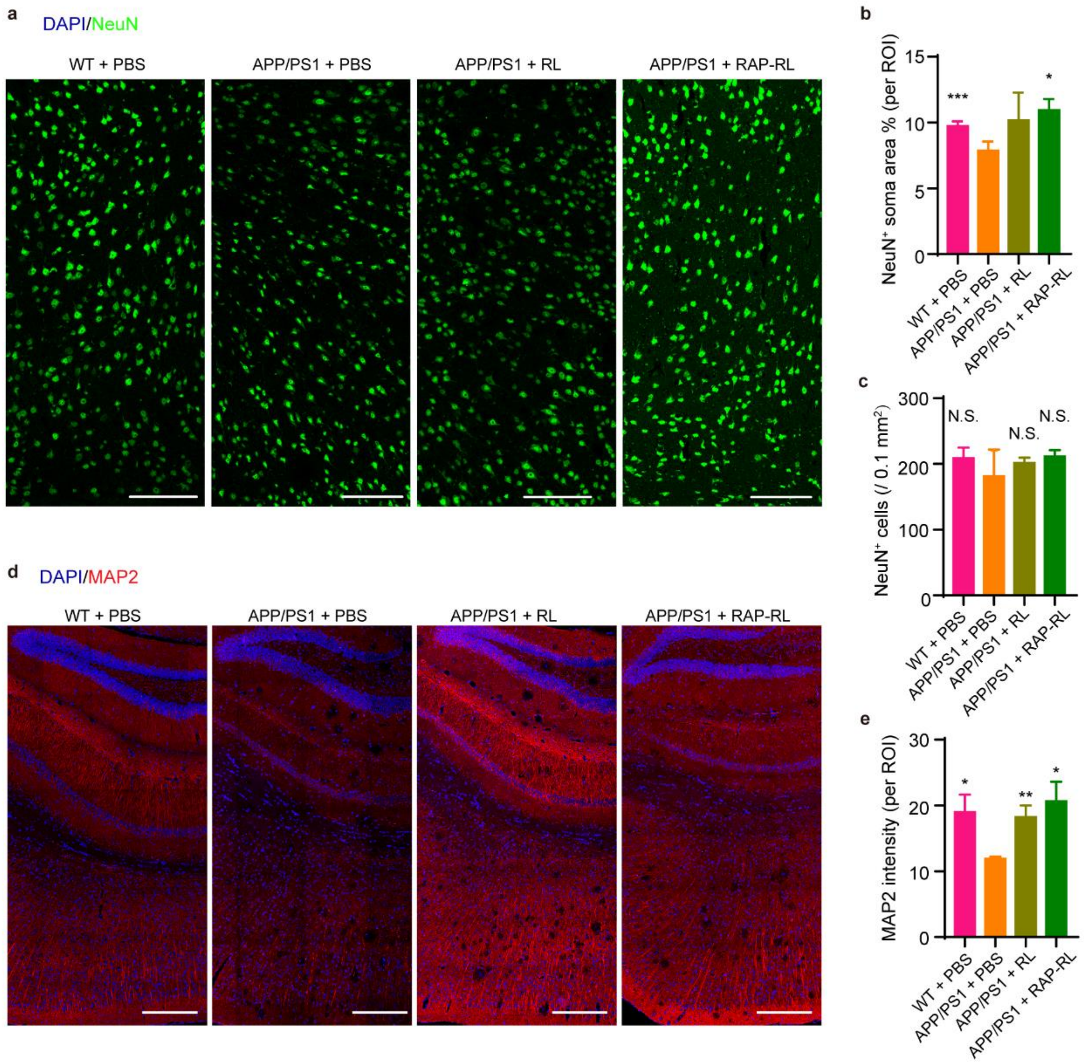

Figure S11. RAP-RL ameliorates neuron soma shrinking and dendrite structure loss in

APP/PS1 mice. Thirteen-month-old male APP/PS1 mice were treated with RAP-RL or RL for four weeks with the age-match WT and APP/PS1 mice treated with PBS as the normal and negative control, respectively. n=3-4. a) Immunofluorescent analysis of neuron in the cortex and hippocampus. Anti-NeuN labels neuron soma, DAPI labels cell nuclei. Scale bars, 100 μm. b) Quantification of $NeuN^+$ cells number and c) $NeuN^+$ area in the cortex. d) Immunofluorescent analysis of dendrite in the cortex and hippocampus. Anti-MAP2 labels dendrite, DAPI labels cell nuclei. Scale bars, 250 μm. e) Quantification of MAP2 fluorescence intensity in the cortex and hippocampus. Data represent the mean ± SEM. *P <0.05, **p <0.01, ***P <0.001. Student's t-test for group comparisons. N.S., not significant.

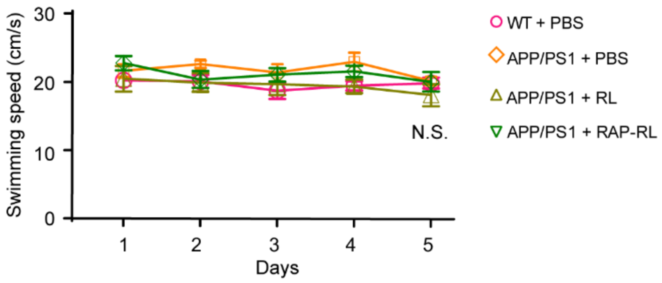

Figure S12. RAP-RL did not change swimming speed in APP/PS1 mice. Ten-month-old female APP/PS1 mice were treated with RAP-RL or RL for four weeks with the age-match WT and APP/PS1 mice treated with PBS as the normal and negative control, respectively. n=8-9. Data represent the mean ± SEM. One-way ANOVA with Tukey's multiple-comparisons test for group comparisons. N.S., not significant.

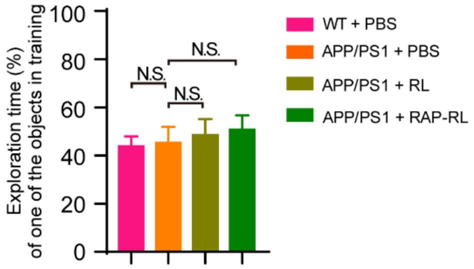

Figure S13. Different groups of animals did not show a preference for either object. Ten-month-old female APP/PS1 mice were treated with RAP-RL or RL for four weeks with the age-match WT and APP/PS1 mice treated with PBS as the normal and negative control, respectively. Data represent the mean ± SEM. One-way ANOVA with Tukey's multiple-comparisons test for group comparisons. n=8-9. N.S., not significant.

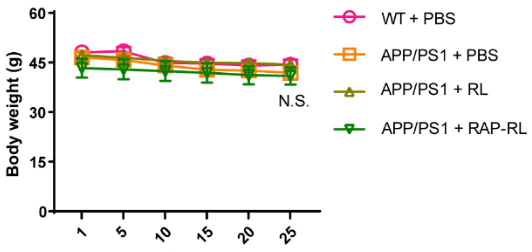

Figure S14. Effect of RAP-RL on body weight of APP/PS1 mice. Ten-month-old female APP/PS1 mice were treated with RAP-RL or RL for four weeks with the age-match WT and APP/PS1 mice treated with PBS as the normal and negative control, respectively. Data represent the mean ± SEM. One-way ANOVA with Tukey's multiple-comparisons test for group comparisons. n=8-9. N.S., not significant.

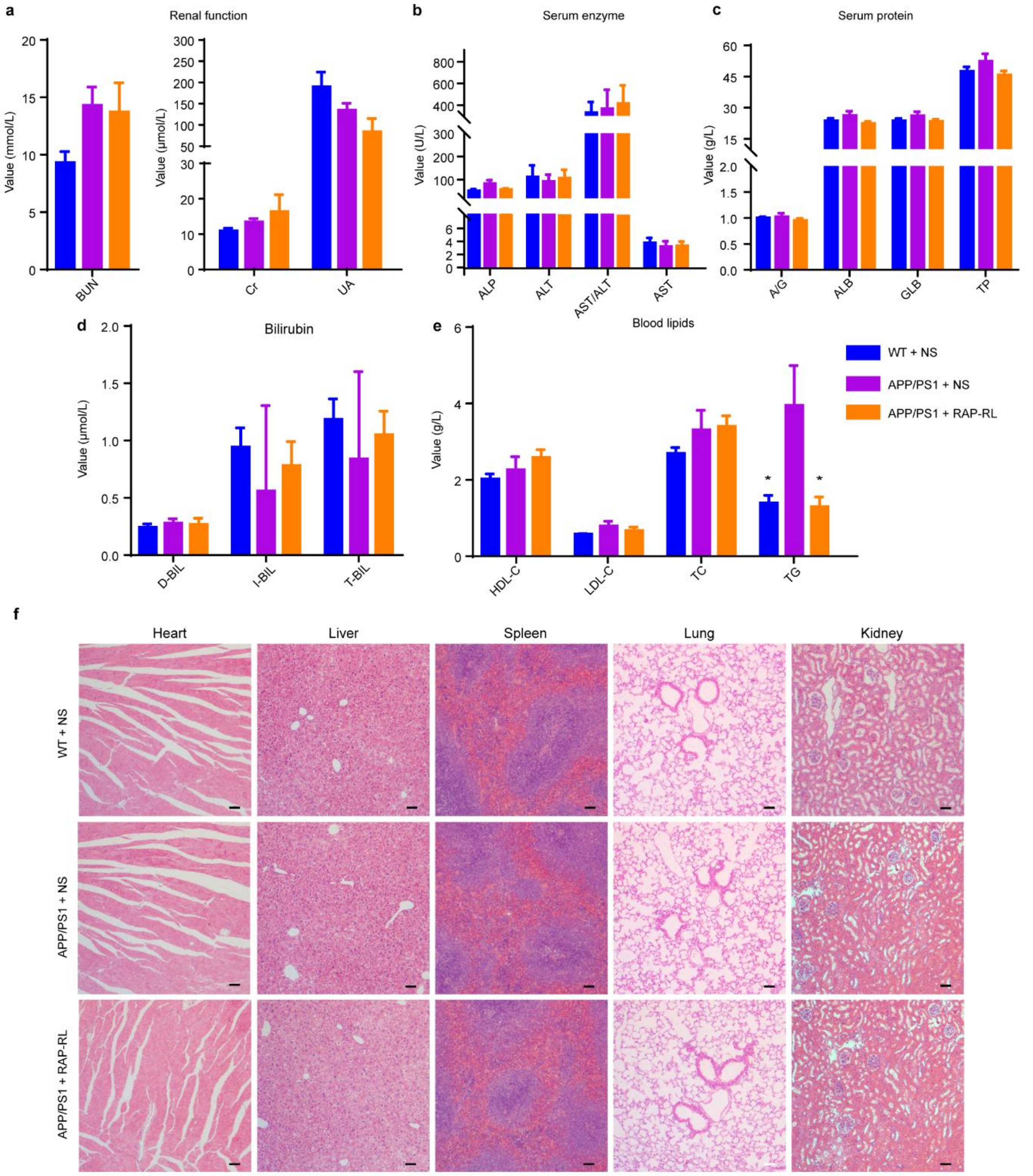

Figure S15. Evaluation of the safety of RAP-RL on APP/PS1 mice. a) RAP-RL showed no effect on renal function (blood urea nitrogen, BUN, Serum creatinine, Cr, uric acid, UA). b-d, RAP-RL showed no substantial differences on the liver injury markers. b) Quantification of alkaline phosphatase (ALP), alanine aminotransferase (ALT), aspartate aminotransferase (AST) and AST/ALT in WT, APP/PS1 and RAP-RL treated APP/PS1 mice. c) Quantification of albumin (ALB), globulin (GLB), ALB/GLB (A/G) and total protein (TP) in WT, APP/PS1 and RAP-RL treated APP/PS1 mice. d) Quantification of direct bilirubin (D-BIL), indirect

bilirubin (I-BIL) and total bilirubin (T-BIL) in WT, APP/PS1 and RAP-RL treated APP/PS1 mice. e) Quantification of high density liptein cholesterol (HDL-C), low density liptein cholesterol (LDL-C), total cholesterol (TCH) and triglyceride (TG) in WT, APP/PS1 and RAP-RL treated APP/PS1 mice. In a, b, c, d, n = 5-7 mice per group. f) Hematoxylin and eosin staining of the major organs. Scale bar, 50 μm. Data represent the mean ± SEM. *P<0.05, One-way ANOVA with Tukey's multiple-comparisons test for group comparisons.

Experimental Section

Animals. APP/PS1 mice were purchased from the Model Animal Research Center of Nanjing University. The animals were maintained in the specific pathogen-free animal facility at 22 ± 2°C on a 12 h light-dark cycle with free access to food and water. All animal experiments were approved by the Animal Experimentation Ethics Committee of Shanghai Jiao Tong University School of Medicine.

Preparation of RAP-RL. Firstly, dissolve DMPC (3.6 mg, Avanti) and GM1 (0.4 mg, Avanti) into a mixed solution of methanol/chloroform (1:2, v/v). After that, the mixed solution was dried under a high vacuum at 37□, the lipid film was rehydrated with 3.6 mL of PBS buffer (0.01 M, pH 7.4) to form GM1-DMPC liposome. To prepare RL, human recombination ApoE3 (0.5 mg, Pepro Tech) was added into the liposome solution and incubated at 37℃ for 36 h. To prepare RAP-RL, αRAP (FAEKFKEAVKDYFAKFWD-GSG-ELKVLMEKEL, GL Biochem Ltd., purity > 95%) (2 mg/mL), dissolved in a mixed solution of acetonitrile/deionized water (1:2, v/v), was added at the molar ratio 1:30 (αRAP: DMPC) and incubated at 37℃ for 24 h. After that, ApoE3 (0.5 mg) was added and further incubated at 37℃ for 36 h.

Characterization of RAP-RL. A Zetasizer Nano-ZS90 system (Malvern Instruments, U.K.) equipped with a He-Ne laser (4 mW, 633 nm and a detector angle of 90°) was applied to measure particle size distribution and zeta potential of RAP-RL. TEM (JEOL, JEM-1400,

Japan) and cyro-EM (FEI Tecnai F20 cryo-electron microscope, Holland) were applied to characterize the morphology and size of RAP-RL. To prepare TEM samples, RAP-RL (0.1 mg/ kg DMPC) was negative stained with a 1.5% sodium phosphotungstate solution. A FEI Vitrobot (FEI, Holland) was applied to prepare cryo-electron microscopy samples and the frozen hydrated samples were analyzed at 200 kV at -180℃.

Cell culture. bEnd.3 cells were purchased from the Type Culture Collection of the Chinese Academy of Sciences, Shanghai, China. And cells were grown in DMEM medium supplemented with 10% FBS, 1% non-essential amino acids, 100 units/mL penicillin, and 100 μg/mL streptomycin, and in a 5% CO_2 humidified atmosphere at 37℃.

Quantification of cellular uptake of DiI-RAP-RL. bEnd.3 cells were cultured in a glass-bottom 96-well plate (In Vitro Scientific) at the density of 5×10^3 cells/well in DMEM with 10% FBS. Twenty-four hours later, the cells were treated with DiI-RAP-RL (5 μg/mL DMPC) in DMEM for 6 h at 37℃. For uptake inhibition, FPS-ZM1 (RAGE inhibitor, Selleck Chemicals; 5 μM) was applied to pretreat bEnd.3 cells. One hour later, DiI-RAP-RL was added and incubated for 3 h. Then, the cells were washed for 3 times and fixed with formaldehyde solution (3.7%, v/v) for 15 min at 37℃. Finally, the cell nuclei were stained with DAPI. The amount of DiI-RAP-RL internalized by bEnd.3 cells was analyzed via an HCS instruments (Thermo Scientific Cellomics, Thermo, USA) as described previously[1].

Assessment of claudin-5 and ZO-1 in vitro. bEnd.3 cells were seeded at a density of 5.0×10^3 cells/well onto 24-well in 96-well plate (Corning) in DMEM with 10% FBS. After cells grew into a confluent monolayer, bEnd.3 cells were pretreated with $A\beta_{1-42}$-oligomer (5 μM) for 18 h, and then incubated with RAP-RL (containing 6 μg/mL DMPC), RL (containing 6 μg/mL DMPC) or α-RAP (0.92 μg/mL) for 18 h. Finally, Cultured bEnd.3 were fixed with 4 % formaldehyde for 15 minutes at 37°C and were incubated with 5% normal goat serum in PBS for 30 minutes at room temperature to block the nonspecific binding of antibodies. For

claudin-5 and ZO-1 staining, see the steps in "*Immunohistochemistry and immunofluorescence*". The images were acquired using a Leica TCS SP8 laser scanning confocal microscope.

Binding analysis of RAP-RL on human brain slices. Human brain slices from three AD patients and three controls with neither of AD nor other mixed dementia were included in present study. The AD patients consist of one male and two females ranging from 72 to 89 years old, and the controls consist of one male and two females ranging from 80 to 84 years old, without history of cerebrovascular diseases or other types of dementia. Postmortem brain slices were obtained from the Human Brain Bank of Peking Union Medical College (PUMC). After blocking, the sections incubated with DiI-RAP-RL (10 μg/mL DMPC) for 3.5 h. For GLUT1 staining, see the steps in "*Immunohistochemistry and immunofluorescence*". DAPI was used for nuclei staining. The images were acquired using a Leica TCS SP8 laser scanning confocal microscope. The present study was approved by the Institutional Review Board of the Institute of Basic Medical Sciences, Chinese Academy of Medical Sciences (Approval Number: 009-2014,031-2017).

Co-immunoprecipitation analysis. The binding ability of RAP-RL to RAGE was evaluated in APP/PS1 mice plasma as previously reported[2]. Firstly, we prepared RAP-RL and RL with fluorescent probe DiI (2% of DMPC, w/w). Then, 120 μL RAP-RL/RL (80 μg DMPC / mL) and 40 μL human recombination RAGE protein (Biovision, Cat# 4271-50, 200 μg/ mL) were mixed in APP/PS1 mice plasma and incubated at 4°C overnight. After that, the bait protein RAGE antibody (8 μg/mL) was added to capture the RAGE:RAP-RL or RAGE:RL complex. Protein G agarose (100 μL) was applied to bind RAGE antibody for 2 h. Finally, the fluorescent intensity of pulled down RAGE antibody: RAGE:RAP-RL or RAGE antibody:RAGE:RL complex was measured at Ex/Em 522/560nm.

Drug treatment. For in vivo RAP-RL treatment, 9-month-old female APP/PS1 mice were divided into three groups randomly, and daily intravenously given with PBS, RL (0.34 mg/kg

DMPC) and RAP-RL (0.34 mg/kg DMPC) for 4 weeks, respectively. Twelve-month-old male APP/PS1 mice were randomly divided into three groups randomly and daily intravenously given with PBS, RL (1.34 mg/kg DMPC) and RAP-RL (1.34 mg/kg DMPC) for 4 weeks, respectively. The age-match wild type littermates treated with PBS served as the normal control. For in vitro treatment, RAP-RL or RL stock solution was filtered with a 0.22 μm membrane and diluted in DMEM to the designed concentration.

In vivo multi-photon imaging. The mice were anesthetized with i.p. injection of ketamine (100 mg/kg) and midazolam (5 mg/kg). Then, in the parietal bone, taking the following coordinates (1.8 mm posterior to the bregma, 1.8 mm lateral to the midline) as the center, a cranial window (2.5-mm-diameter, 7 mm^2) was opened over the cerebral cortex using a high-speed drill (0.6 mm diameter). After removing the circular bone fragment, a thin grass was carefully placed above the cranial window and fixed with dental cement. Before imaging, the cranial window was covered with artificial CSF. A water-immersion objective (25 ×, NA 1.05; Olympus, Japan) was used to image the cerebral vessels or amyloid plaques. A two-photon imaging system (FVMPE-RS, Olympus, Japan) equipped with a Mai Tai laser (Spectra-Physics) and an upright microscope was applied for imaging. The images were acquired at the excitation 900 nm.

For analyzing diameter of the cortical micro-vessels, we injected rhodamine-dextran (70,000 Da, Sigma-Aldrich; 0.15 mL of 20 mg/mL) intravenously into APP/PS1 mice. The images of the cerebral vessels were captured from 100 μm to a depth of 500 μm below the surface of the cortex. The scanning area was 509 × 509 μm with a resolution of 1024 ×1024. The z step is 1 μm.

For visualizing amyloid plaques, methoxy-XO4 was dissolved in ethanol (Tocris Bioscience, Bristol, UK) and i.p. injected into APP/PS1 mice (3 mg/kg) 24 h prior to imaging. Before amyloid imaging, rhodamine-dextran (70,000 Da; Invitrogen, MA; 0.15 mL of 20

mg/mL) was intravenously injected to indicate the blood vessels. Similarly, the images were captured from 100 μm to a depth of 500 μm below the surface of the cortex. The scanning area was 509 × 509 μm with a resolution at 512×512. The z step is 2 μm.

Preparation of $A\beta_{1-42}$ oligomer. $A\beta_{1-42}$ oligomer was prepared and characterized as described in our previous work[1]. $A\beta_{1-42}$ (Invitrogen, 1 mg) was firstly dissolved in 1 mL hexafluoroisopropanol (HFIP), the solvent was then evaporated. The peptide film was resuspended in dimethyl sulfoxide (DMSO) to make a 5 mM solution, diluted in PBS (0.001 M, PH 7.4) to a 100 μmol/L final concentration and incubated overnight at 4°C before use.

$A\beta_{1-42}$ clearance analysis. Label $A\beta_{1-42}$ oligomer with ^{125}I through the Iodogen iodination method as previously described[1]. Briefly, the reaction was initiated by the addition of 10 μg of $A\beta_{1-42}$, 30 μL of PBS (pH 7.4, 0.1 M) and 1.23 mCi of $Na^{125}I$ in an iodogen-coated tube. After reacting for 5 min, the radioiodination mixture was subjected to Sephadex G-10 column for separation to discard free radioiodine. The ^{125}I-$A\beta_{1-42}$ was diluted in artificial CSF and the final radioactivity of ^{125}I-$A\beta_{1-42}$ solution is 0.046 μCi/μL. APP/PS1 mice were anesthetized and secured with a stereotaxic frame. ^{125}I-$A\beta_{1-42}$ (0.28 ng per mice) was injected intra-hippocampally through a microsyringe in 3 min, according to the coordinates. Thirty minutes later, the mice were sacrificed, and the whole brain was harvested and weighed. Analysis the radioactivity of whole brains was performed via a γ-counter. The intact $A\beta_{1-42}$ remained in the brain was assayed after homogenizing and precipitating brain using 3-fold weight of cold TCA.

For calculations of clearance rates. The analysis of ^{125}I-$A\beta_{1-42}$ disappearance from the brain was as reported[3]. The percentage of radioactivity remaining in the brain after microinjection was determined as % Recovery in brain = $100 \times \frac{\mathrm{Nb}}{\mathrm{Ni}}$ [1]

$$100 \times \left(\frac{\mathrm{Nb}}{\mathrm{Ni}}\right) = 100\% \times \frac{\text{amount of TCA precipitated remaining A}\beta\text{ in brain}}{\text{amount of A}\beta\text{ injected}}$$

MRI. CBF was measured via MRI experiment with a 7.0 T scanner (Bruker, BioSpec70 /

20USR), with a 20 cm warm bore diameter and high performance actively shielded gradient system. For the analysis, the mice were anesthetized with 1.0-1.5% isoflurane, maintained on a waterbed to keep body temperature at 37˚C and monitor their respiratory rates continuously with a vital sign monitoring system. During the experiment, the mice's respiratory rates were kept at 50-60 / min. The mice were fixed with two ear bars and a bite bar. First, anatomical T2-weighted MR images were performed to determine the arterial spin labeling (ASL) location. We applied the relaxation enhancement (RARE) sequence echo time = 35 ms, repletion time = 2000 ms, field of interest (FOV) = 20 mm × 20 mm, and matrix = 256 × 256) to image the whole hippocampus in 10 slices at thicknesses of 0.5 mm. Positioning the appropriate hippocampus at thickness of 1 mm based on RARE images for ALS. The ASL parameters were as follows: FOV = 20 mm x 18 mm; slice thickness = 1 mm; matrix = 128 × 115; number of excitations = 1. The scan time lasted for 260 s. The obtained ASL images were analyzed as described previously[4], using the workstation software ParaVision 6.0 (Bruker Corporation) to generate CBF images. For CBF values determination, brain regions-of-interest (ROI) boundaries were drawn manually according to anatomical T2- weighted images with the same slice location displayed concurrently for reference. Then, the ROIs were overlaid onto the CBF map with an accurate boundary alignment. The mean CBF of each brain was calculated.

SPR analysis. SPR analysis was performed by using a Biacore T200 system (GE Healthcare, USA) with CM5 sensor chips. Human recombination RAGE protein (Biovision, Cat# 4271-50) was immobilized by amine coupling onto the chips as previously described. The parallel flow cells non-immobilized were used as the reference channel. Various concentration of RAP-RL (diluted in 0.01 M PBS, pH 7.4 plus 0.05% tween 20) was injected into the flow system at a flow rate of 30 μL/min. Dissociation lasted for 6 min. Binding curves were analyzed via a 1:1 Langmuir binding model using BIA evaluation program (GE Healthcare).

Immunohistochemistry and immunofluorescence. For most immunohistochemistry and

immunofluorescence analysis, sample preparation was performed as previously described[5]. After heat perfusion, the brain of the animals was collected and fixed in 10% formalin solution, embedded in paraffin, and sectioned into 4 μm. For DiI-RAP-RL and RAGE/GLUT1 colocalization analysis, the brains were post-fixed overnight in 4% paraformaldehyde, and sectioned at 30 μm with a Leica VT1000S vibratome (Leica).

For immunohistochemistry analysis, the brain slices were pretreated as previously[1]. Then, the brain slices were incubated overnight at 4°C with primary antibody solution diluted in staining solution (10% normal goat serum and 0.5 % Triton X-100 in PBS). Primary antibodies included mouse anti-claudin 5 (1:500, Cat#35-2500, Invitrogen), mouse anti-Aβ 6E10 (1:250, Cat#SIG-39320, Covance), rabbit anti-GLUT1 (1:500, Cat#ab115730, Abcam). The secondary antibodies used were: Alexa Fluor 488-conjugated donkey anti-rat IgG (1:20000, Cat#A-21206, Invitrogen), Alexa Fluor 568-conjugated donkey anti-rabbit IgG (1:20000, Cat# A10042, Invitrogen), Alexa Fluor 488-conjugated donkey anti-mouse IgG (1:20000, Cat# R-37114, Invitrogen), Alexa Fluor 568-conjugated donkey anti-mouse IgG (all from Invitrogen) (1:20000, Cat# A-10037, Invitrogen).

For the immunofluorescence analysis, the brain slices were incubated with primary antibody solution diluted in mixed solution (10% normal goat serum and 0.2 % Triton X-100 in PBS) overnight at 4°C. Primary antibodies included rabbit anti-RAGE (1:200, Cat#ab3611, Abcam), rabbit anti-GLUT1 (1:200, Cat#ab115730, Abcam), mouse anti-CD13 (1:300, Cat#sc-13536, SANTA CRUZ BIOTECHNOLOGY), rabbit anti-α-SMA (1:100, Cat#ab5694, Abcam), mouse anti-AQP4 (1:200, Cat#ab9512, Abcam), rabbit anti-Iba-1 (1:100, Cat#ab178847, Abcam), and mouse anti-Aβ 6E10 (1: 50, Cat#SIG-39320, Covance), mouse anti-MAP2 (1:200, Cat#ab11267, Abcam), mouse anti-claudin-5 (1:200, Cat#35-2500, Invitrogen). After that, samples were washed in PBS for 3 times, and stained with secondary antibodies (Invitrogen) for 3 h at room temperature. Secondary antibodies including donkey anti-rabbit Alexa Fluor 568 (Cat#A10042), goat-anti-mouse Alexa Fluor 488 (Cat#A-10680)

(1:2000) were obtained from Life tech.

DAPI was used for nuclei staining. The images were acquired using a Leica TCS SP8 laser scanning confocal microscope.

Image analysis. Immunohistochemical images were captured at ×40 magnification with a Nikon Eclipse E200 mounting the CCD Camera HTC1600, at a resolution of 2,320 × 1,740. Immunofluorescence images were taken at ×40 magnification with Leica TCS SP8 laser scanning confocal microscope, with a resolution of 1,024 × 1,024 or 512 × 512. Quantitative analysis was performed with Image J Fiji. Before the analysis, all the images were first converted to 8-bit, black and white images, and then subjected to threshold processing according to the signal density difference between positive area and background. A detailed analysis method is available in supplementary materials.

Detailed image quantitative analysis.

1. Quantification of the CD13 and AQP4 coverage

The coverage of CD13 and AQP4 was analyzed as described previously[5]. Firstly, CD13 and AQP4 signals were reconstructed as maximum Z-projections of 5-μm-thick volumes. The selected cerebral vessels (diameter ≤ 10 μm) were subjected to threshold processing (use the same threshold parameter for the same evaluation indicator) and then analyzed through the ImageJ Fiji colocalizations plugin. Pericyte/AQP4 coverage was indicated by the percentage (%) of CD13/AQP4-positive area covering tomato lectin-positive capillary area per field (278 μm × 278 μm). Six to eight fields in the cortex region and CA1/CA3 region of each animal were included for the quantification.

2. Analysis of Aβ plaque-associated microglia

After threshold processing, plaques (diameter is between 30 to 40 μm) and the surrounding microglia were analyzed. Microglial-Aβ colocalization was indicated by the ratio of the 6E10 and Iba1 double-positive area to the 6E10-positive area. Analyze particles plugin in Image J Fiji was applied for quantifying number of Aβ-related microglia.

3. Quantification of specific area density

For capillary density, claudin-5 density, GFAP area density, Iba1 area density, NeuN positive neuronal density and MAP2 intensity quantification, the cortex region and CA1/CA3 region of each animal were included for the quantification. The total fields included for the quantification were illustrated in the figure. For capillary density analysis, images without inappropriate vessels (diameter > 10 μm) were used.

After threshold processing (use the same threshold processing standard for the same evaluation indicator), the signal of area density was calculated using analyze particles plugin. The parameters "% Area" and "Mean intensity" were used for statistical analysis.

4. Quantification of microvascular diameter

Vessel analysis plugin in Image J Fiji was used for quantification according to its manual. Before the analysis, Wand tool was used to removed inappropriate vessels (diameter > 10 μm).

5. Quantification of perivascular Aβ deposition

For immunochemical data, after threshold processing, polygon selections tool was used to circle the micro-vessel area as the ROI. The level of perivascular Aβ deposition was reflected by the pixel-based area of Aβ (6E10) signal per unit ROI.

For immunofluorescence data, after threshold processing, Image J Fiji colocalizations plugin was used to calculate the integrated signal density of each image. The level of perivascular Aβ deposition was indicated by the percentage (%) of methoxy-XO4-positive Aβ area accounting for the rhodamine-dextran positive cerebral vessels area.

6. Quantification of Aβ volume ratio

Aβ volume ratio represents the proportion of the reconstructed Aβ in the given multi-photon microscopy 3D images. For the analysis, 3D objects counter plugin in Image J Fiji was used for the quantification of Aβ volume ratio according to its manual.

Analysis of LSCI. CBF response to electrical stimulation of whisker was performed as

previously described using LSC[6]. After anesthesia, the skin over the skull was cut open to expose the right side of the skull. A customized electrode was applied. The cathode was implanted at the left hiatus infraorbitalis (IO), and the anode was implanted into the left masticatory muscles. The light source (a near infrared laser diode) and camera (CMOS camera) are the same as the previous experimental conditions[6]. The electrical stimulation condition was 0.5 mA [amplitude], 5 Hz [frequency], 1 ms [pulse width], and stimulation time was 2 s. Six consecutive trials were applied. At the beginning of each trial, the first 4 s is for baseline and stimulate for 2 s followed by 14 s for recovery. The interval time of each trail is 60 s. For presentation in Figure 6i, the data were normalized such that the average basal relative CBF change was set to zero and the peak set to 1[7]. Subsequently, linear parametric functions were fitted to the relative CBF change of each mouse from onset of stimulation to the peak CBF point. The slope of CBF to time was calculated to determine the CBF response rate upon stimulation.

Enzyme linked immunosorbent assay (ELISA). For determining the protein levels in the CNS, cortices, and hippocampus were isolated from the brain firstly and homogenized in radioimmunoprecipitation assay (RIPA) buffer (containing 1 mM EDTA and 10 mM PMSF), and then centrifuged at 12,000 rpm for 20 min to obtain the supernatant. Then, we applied a bicinchoninic acid (BCA) assay to evaluate protein levels in the supernatants. The levels of IL-6, and TNF-α (Multisciences Biotech, Hangzhou, China) were quantitatively measured by ELISA kit according to the manufacturer's instructions.

MWM test. The MWM test was performed as previously described[1]. Training procedure lasted for five days. The mice were trained 4 times daily from 4 positions. If the mice reached the platform within 90 s, they stay there for 10 s. Otherwise, they were guided to the platform and stayed for 30 s. On the probe trail day, place the mice in the pool without the platform from the two different position adjoining the platform by order. All analysis was recorded by a tracking system (Shanghai Jiliang Software Technology).

NOR test. The NOR test equipment contains an acrylic cube (35 cm × 35 cm) with a white bottom. The test procedure is divided into phases: habituation, familiarization, and test phase. On habituating day, each mouse was gently placed into the open-field area without objects and allowed to explore the field freely. Five minutes later, the mouse was transferred from the open filed to its facility. Clean the area with alcohol to eliminate the odor and test the next mouse. On the day of training, two identical objects were located in different positions in the open filed. A single mouse was placed in the acrylic cube with its back to the object for 5 min. On the testing day, one of the identical objects was replaced by a novel one. The mice were placed in open-field area one by one and stay for 5 min.

$$\text{Discrimination Ratio} = \frac{\text{Time interaction with novel object}}{\text{Time interaction with sample object} + \text{Time interaction with novel object}} \times 100\%$$

Detection of d9-RAP-RL. We used methanol to extract d9-RAP-RL remaining in the brain homogenates and selected 13:0 PC as the internal standard. Equip the API-4000 mass spectrometer with an electrospray ionization source and operate it in the positive-ion mode. A C18 column, 3.0 μm, 4.6 mm × 100 mm (Agela Technology, China) was applied to perform chromatography with the mobile phase consisted of 0.1% formic acid and 5 mM ammonium formate in water (A) and isopropanol (B) at the flow rate of 0.5 mL/min (A:B, 85:15). The ionization source parameters: turbo heater temperature 600°C, turbo heater gas (GAS2) 60 psi, nebulizer gas (GAS1) 60 psi, curtain gas 20 psi, collision gas (CAD) 4 psi, ion spray voltage 4500 v. The collision energy (CE) varied in the range of 41 V, declustering potential (DP) was 170 V. Multiple reaction monitoring (MRM) mode was conducted to detect the ions, monitoring the transition of the m/z 687.5/193.1 for d9-DMPC and 650.5/184.1 for 13:0 PC (as internal standard), respectively.

Statistical analysis. In the same experiment, sets of mice with the same age and sex were kept in the same conditions and randomly allocated to the different experimental groups. Prism 7.0 (GraphPad Software) was used for the statistical analyses. Data are presented as

mean ± SEM., with $P < 0.05$ considered statistically significant. Group differences were assessed by unpaired Student's *t*-test, one-way analysis of variance (ANOVA) or two-way ANOVA followed by Tukey's multiple comparisons test as mentioned in the text and figure legends.

References

[1] M. Huang, M. Hu, Q. Song, H. Song, J. Huang, X. Gu, X. Wang, J. Chen, T. Kang, X. Feng, D. Jiang, G. Zheng, H. Chen, X. Gao, *ACS Nano* **2015**, *9*, 10801.

[2] Q. Song, M. Huang, L. Yao, X. Wang, X. Gu, J. Chen, J. Chen, J. Huang, Q. Hu, T. Kang, Z. Rong, H. Qi, G. Zheng, H. Chen, X. Gao, *ACS Nano* **2014**, *8*, 2345.

[3] M. Shibata, S. Yamada, S. R. Kumar, M. Calero, J. Bading, B. Frangione, D. M. Holtzman, C. A. Miller, D. K. Strickland, J. Ghiso, B. V. Zlokovic, *J Clin Invest* **2000**, *106*, 1489.

[4] Y. Guo, X. Li, M. Zhang, N. Chen, S. Wu, J. Lei, Z. Wang, R. Wang, J. Wang, H. Liu, *Mol Med Rep* **2019**, *19*, 3045.

[5] R. D. Bell, E. A. Winkler, A. P. Sagare, I. Singh, B. LaRue, R. Deane, B. V. Zlokovic, *Neuron* **2010**, *68*, 409.

[6] B. Bo, Y. Li, W. Li, Y. Wang, S. Tong, *IEEE Trans Biomed Eng* **2019**, *66*, 1372.

[7] K. Kisler, A. R. Nelson, S. V. Rege, A. Ramanathan, Y. Wang, A. Ahuja, D. Lazic, P. S. Tsai, Z. Zhao, Y. Zhou, D. A. Boas, S. Sakadzic, B. V. Zlokovic, *Nat Neurosci* **2017**, *20*, 406.
